# Hydrophobic and Hydrophilic Extractives in Norway Spruce and Kurile Larch and Their Role in Brown-Rot Degradation

**DOI:** 10.3389/fpls.2020.00855

**Published:** 2020-06-30

**Authors:** Sophie Füchtner, Theis Brock-Nannestad, Annika Smeds, Maria Fredriksson, Annica Pilgård, Lisbeth Garbrecht Thygesen

**Affiliations:** ^1^Department of Geoscience and Natural Resource Management, University of Copenhagen, Copenhagen, Denmark; ^2^Department of Chemistry, University of Copenhagen, Copenhagen, Denmark; ^3^Laboratory of Wood and Paper Chemistry, Johan Gadolin Process Chemistry Centre, Åbo Akademi University, Turku, Finland; ^4^Faculty of Engineering, Division of Building Materials, Lund University, Lund, Sweden; ^5^Wood Research Munich, Technical University of Munich, Munich, Germany; ^6^Research Institutes of Sweden (RISE), Gothenburg, Sweden

**Keywords:** extractives, brown-rot, spruce, larch, durability, moisture content, heartwood, GC-MS

## Abstract

Extractives found in the heartwood of a moderately durable conifer (*Larix gmelinii var. japonica*) were compared with those found in a non-durable one (*Picea abies*). We identified and quantified heartwood extractives by extraction with solvents of different polarities and gas chromatography with mass spectral detection (GC-MS). Among the extracted compounds, there was a much higher amount of hydrophilic phenolics in larch (flavonoids) than in spruce (lignans). Both species had similar resin acid and fatty acid contents. The hydrophobic resin components are considered fungitoxic and the more hydrophilic components are known for their antioxidant activity. To ascertain the importance of the different classes of extractives, samples were partially extracted prior to subjection to the brown-rot fungus *Rhodonia placenta* for 2–8 weeks. Results indicated that the most important (but rather inefficient) defense in spruce came from the fungitoxic resin, while large amounts of flavonoids played a key role in larch defense. Possible moisture exclusion effects of larch extractives were quantified via the equilibrium moisture content of partially extracted samples, but were found to be too small to play any significant role in the defense against incipient brow-rot attack.

## 1. Introduction

Wood used as building material in an outdoor environment (i.e., European Standard EN 335-1:2006 use class 3–4) is frequently exposed to high relative humidity or wetting, highly increasing the risk of degradation by wood-degrading micro-organism. The resistance of wood to degradation in such a setting is mainly determined by its inherent durability (natural or artificial) and its moisture sorption properties, apart from environmental factors and design (Brischke et al., 2006; Meyer-Veltrup et al., 2017; Brischke and Alfredsen, 2020). Naturally durable heartwood is formed in the center of the stem of some tree species by deposition of various metabolites in the tissue, called extractives (Rowe, 1989). For the heartwood of many species and all of the sapwood this is not the case, and thus artificial wood protection is needed (Kutnik et al., 2017). In Europe, many of the old wood-preservatives were banned due to their high toxicity, and consequently, research and development focused their efforts on more environmentally benign ways to protect wood (Schultz and Nicholas, 2002; Singh and Singh, 2012). Of the new preservative compounds, many still have restricted use (ECHA, 2020), and thus further developments are necessary. One approach is to achieve a better understanding of the mechanisms underlying natural durability in trees, and the current study aims to contribute to this.

In northern Europe, wood products from conifers are widely used for construction purposes. Most of these are susceptible to degradation by brown-rot fungi, which are known to cause a rapid loss of strength already at an early stage of degradation (Bader et al., 2012; Arantes and Goodell, 2014; Wagner et al., 2015). Brown-rot fungi are widely spread, cellulose-degrading fungi, currently believed to start their attacks employing non-enzymatic oxidative degradation, followed by an enzymatic stage (Bader et al., 2012; Arantes and Goodell, 2014; Zhang et al., 2016). Non-enzymatic oxidative degradation of the cell wall relies on secretion and diffusion of low molecular weight substances and metal ions into the cell wall, where they react to create radicals that disrupt the cell wall polymers. The success of this process depends heavily on the presence of water in and around the cell wall, but also on the amount and mechanisms of extractives present (Schultz and Nicholas, 2002; Jebrane et al., 2014). In this work, we chose to compare Kurile larch (*Larix gmelinii var. japonica*), a moderately durable conifer (Scheffer and Morrell, 1998; Bergstedt and Lyck, 2007; Metsä-Kortelainen and Viitanen, 2009), to non-durable Norway spruce (*Picea abies*) (Scheffer and Morrell, 1998; Metsä-Kortelainen and Viitanen, 2009) in terms of their extractive composition and their susceptibility to the brown-rot fungus *Rhodonia placenta* after various extraction procedures. Their similar xylem anatomy should provide similar geometrical conditions to the fungus, and accentuate differences arising due to their extractive composition.

Extractives belong to many different chemical groups, and may be divided into more hydrophilic and more hydrophobic types (Giwa, 1973; Willför et al., 2003a, 2006a). Both types are found in the tree species studied here.

One role of the more hydrophobic extractives is possibly to repel water, as the amount of moisture in the wood is critical for the fungus' successful establishment (Meyer and Brischke, 2015; Brischke et al., 2017). For instance, it has been suggested that a certain cell wall moisture content is needed to form pathways within the cell walls to allow diffusion of the fungal low molecular weight substances (Zelinka et al., 2015; Hunt et al., 2018). Hydrophobic molecules, such as those found in the oleoresin of conifers, are good candidates for such functionality by decreasing the wettability of the cell wall (Eberhardt et al., 1994; Harju et al., 2002; Nzokou and Kamdem, 2004; Belt et al., 2017; Sjökvist et al., 2018). Oleoresin is present throughout the xylem, but its composition varies within the stem and changes upon injury or infection (Ekman, 1980; Hillis, 1987; Bohlmann et al., 2000; Holmbom et al., 2008; Mason et al., 2015, p. 73). Its primary components are fats and fatty acids (FAs) and various terpenoids. The non-volatile fraction is mainly composed of diterpenoids (DTs), among which resin acids (RAs) are the most abundant (50–75%) (Higuchi, 1997; Bohlmann et al., 2000; Holmbom et al., 2008). Apart from a role in moisture regulation, the potential of RAs as biocides against multiple insects and micro-organisms was shown in numerous *in vivo* and *in vitro* assays (Micales et al., 1994; Nerg et al., 2004; Keeling and Bohlmann, 2006; Mason et al., 2015), including white rot fungi (Eberhardt et al., 1994), and brown rot fungi (Micales et al., 1994; Nerg et al., 2004). Sterols (STs), which are triterpenoids, have potential as growth retardants in bacteria, and a synergistic role with RAs has been proposed (Burčová et al., 2018). Representative chemical structures of compounds, belonging to these families and relevant for this study, can be viewed in Figure S1.1–7.

Spruce and larch heartwood also contain extractives that are more hydrophilic, mainly lignans (LI) in spruce and flavonoids (FL) in larch (see Figure S1.9–12, Willför et al., 2003a; Gierlinger et al., 2004; Nisula, 2018). Their functions include hindering the creation of (fungal) radicals by chelation of metals needed for the latter, neutralizing occurring radicals (antioxidants) and/or directly harming the fungus (biocide) (Rice-Evans et al., 1996; Willför et al., 2003c; Binbuga et al., 2008; Donoso-Fierro et al., 2009; Chen et al., 2014). Phenolic extractives may additionally play a role in moisture exclusion by bulking of the cell wall (Wangaard and Granados, 1967; Choong and Achmadi, 1991; Nzokou and Kamdem, 2004; Vahtikari et al., 2017). Both lignans (Rowe, 1989; Smith et al., 1989) and flavonoids (Dellus et al., 1997; Ostroukhova et al., 2012) partially form oligo- and polymers in heartwood, and so do the more hydrophobic resin components (Schaller, 2008; Smeds et al., 2016, 2018, p. 164).

Larch is somewhat a special case among the conifers, because it contains up to 30% w/w of a non-structural, hemicellulose-type polysaccharide - arabinogalactan (ArGal). Its amounts increase drastically at the sapwood-heartwood boundary, and fills the lumen of tracheid cells in the heartwood, especially those closer to rays (Côté et al., 1966; Giwa, 1973; Grabner et al., 2005a,b). Experiments show that ArGal has influence on the mechanical properties of larch heartwood (Luostarinen and Heräjärvi, 2013), but its role in fungal degradation, if any, remains controversial (Côté et al., 1966; Gierlinger et al., 2004; Hill et al., 2015).

Wood degrading fungi generally need a minimum water potential in the range of −4 to 0.1 MPa in order to grow (Griffin, 1977; Boddy, 1983; Griffith and Boddy, 1991; Schmidt, 2006), which corresponds to 97–99% relative humidity (RH). Above this range, liquid water accumulates in pits and cell lumina via capillary condensation (Engelund et al., 2013; Fredriksson and Thybring, 2019), while in the hygroscopic range (0 to about 98% RH) water is bound to hydroxyl groups in the cell wall. Although it has been shown that extractives alter the equilibrium moisture content (MC) in the hygroscopic range (Wangaard and Granados, 1967; Choong and Achmadi, 1991; Nzokou and Kamdem, 2004; Vahtikari et al., 2017), to the best of our knowledge, no publications exist on how extractives affect the MC in the over-hygroscopic range. The pressure plate technique allows investigation of the wood's MC in this range by precise regulation of the pressure applied to a water containing, tight cell (Fredriksson and Thybring, 2019). After a long equilibration time, the moisture content is determined gravimetrically. One aim of this study was, to explore whether extractives also influence the over-hygroscopic moisture range in Kurile larch.

The best results for artificial impregnation is obtained by the combination of different functionalities (Schultz and Nicholas, 2002). Even more so, is it important to understand the individual roles of natural extractives, in order to be able to mimic the mechanisms at play when designing novel wood protection systems. Thus, partial removal of extractives of a certain polarity may give insight on their influence on wood durability against fungal degradation, and by assessing their effect on the MC of the wood possible interrelations between moisture and extractives content can be identified. With the aim of understanding the roles of hydrophobic and hydrophilic extractives in degradation and moisture regulation, we developed a multi-step extraction procedure that partially removes more extractives of a certain polarity from wooden sticks. The extractive composition was obtained by gas chromatography coupled to mass spectrometry and flame ionization detection to gain insights on the quantities of molecules present. Additionally, since moisture is a prerequisite for fungal attack, a specific aim of our study was to explore whether semi-selective removal of extractives with different polarity would affect the equilibrium moisture content in Kurile larch, especially in the important but under-explored over-hygroscopic range.

## 2. Materials and Methods

**Figure 1:**
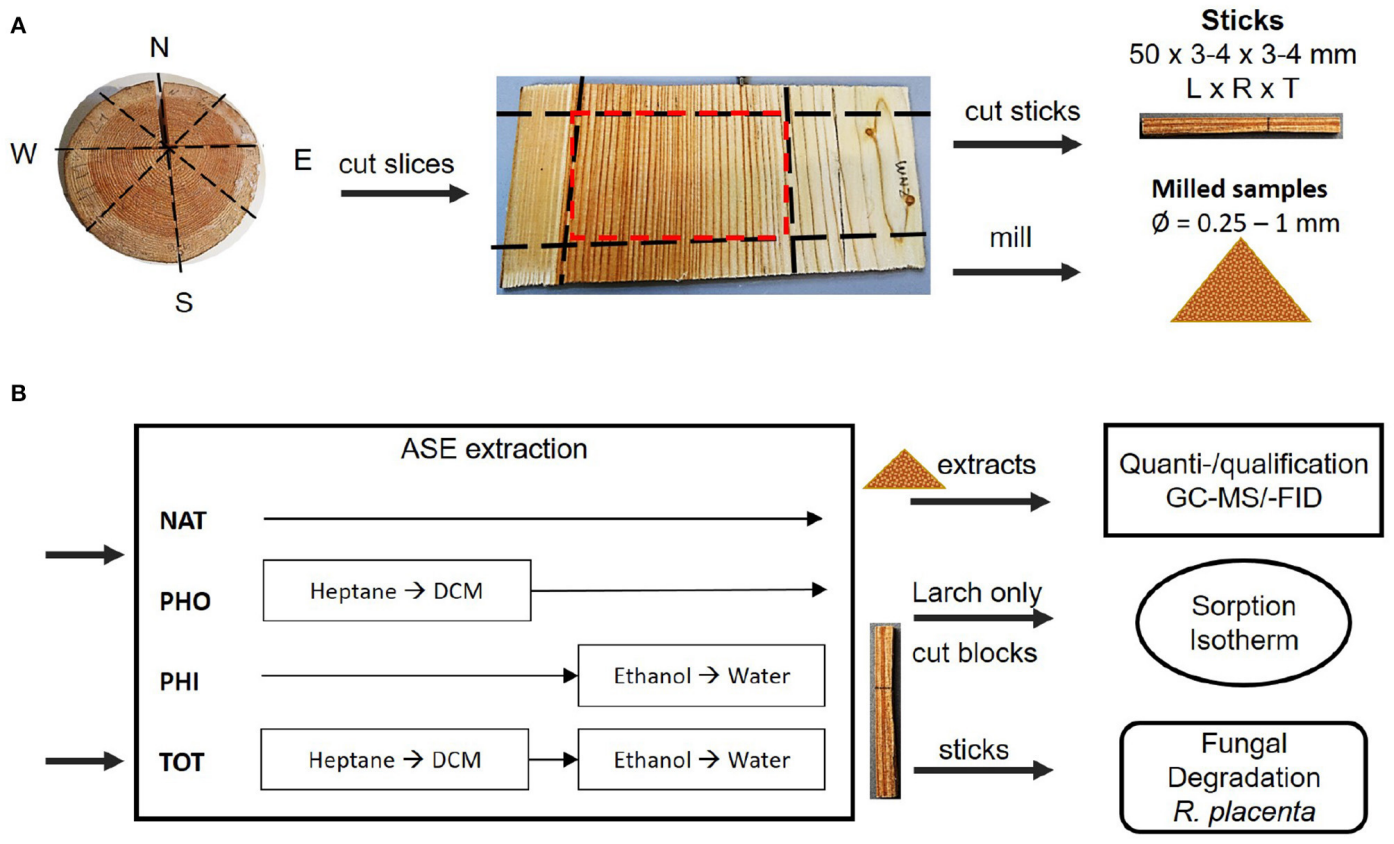
**(A)** Preparation of wooden sticks, exemplified with Larch. The frozen wood disk was cut radially into slices. The mature heartwood (marked in red) was selected and one part was cut into sticks with dimensions 50 × 3–4 × 3–4 mm [longitudinal × radial × tangential]. The other part was milled. **(B)** Sticks and milled samples were extracted with different solvents according to the scheme. The extracts from the milled samples were quantified and identified using GC-MS/-FID. Some larch sticks were made into blocks and used for determination of the sorption isotherm. All extracted and native sticks of both species were subjected to brown-rot degradation. NAT, native; PHO, hydrophobic; PHI, hydrophilic; TOT, total; DCM, dichloromethane.

A summary of procedures and methods used in this work can be viewed in Figure 1.

### 2.1. Wood Sampling and Preparation

Two clones of spruce (*P. abies*, 48 years old) and larch (*Larix gmelinii var. japonica*, 65 years old) were harvested near Hørsholm, Denmark, in the autumn of 2017. The individual trees will be referred to as spruce 1, spruce 2, larch 1 and larch 2. Figure 1A illustrates the sampling process. Disks (about 80 mm thick) were taken at 1.3 m stem height and stored at −20°C within 10 h from sampling. The disks' diameters were 323 and 280 mm for spruce 1 and 2, respectively. For larch 1 and 2 they were 381 and 406 mm, respectively. Using a band saw, 3–4 mm thick slices were radially cut from all around the frozen stem disks. The mature heartwood (red square in Figure 1A) was separated from sapwood and juvenile heartwood. For spruce, 10 growth rings from the pith were considered juvenile (Lindström, 2002). For larch, on average 15 year rings were similarly discarded (Gierlinger and Wimmer, 2004; Luostarinen and Heräjärvi, 2013). The preselected mature heartwood was stored at −80 °C until further processing.

The samples were split into two groups: slices with 3–4 mm width were used to prepare sticks of 50 mm × 3 or 4 mm × 4 or 3 mm (L × W × B), as shown in Figure 1A. The sticks were freeze dried for 30–48 h and categorized into different growth ring patterns, i.e., broad EW + broad LW, thin repeating EW+LW, EW-LW-EW, and LW-EW-LW. These were then distributed evenly into four groups of 13–17 sticks each, so as to avoid possible bias from the radial position in the heartwood, where each stick was comprised of 1–3 annual rings. The rest of the frozen material was made into 10 mm^2^ chips with garden shears. After freeze drying for 24 h, the chips were milled in a Retch ZM100 mill (1 mm sieve), freeze dried again and sieved through a 60 mesh filter, corresponding to a hole size of 250 μm. Before extraction, all samples were placed in a desiccator under vacuum with freshly dried molecular sieves for 2–18 h at room temperature.

### 2.2. Extraction and Further Processing

Extraction of milled samples and sticks was done using the Accelerated Solvent Extractor (Dionex^TM^ ASE^TM^ 350, Thermo Electron A/S, Scientific Instrument Division, 2650 Hvidovre, Denmark). The apparatus works under N_2_ atmosphere, thereby lowering chances of oxidation artifacts possibly arising during extraction. Additionally, 1.38 MPa are applied to the extraction cell, allowing the use of volatile solvents at temperatures above their boiling points.

An extraction procedure was developed, aiming at producing samples where the hydrophobic (PHO) or hydrophilic (PHI) part of extractives had been removed, as well as a total extraction, removing both portions (TOT, Figure 1B). For the TOT extraction a sequence of four solvents was used in the following order: Heptane (anhydrous, 99%, Sigma-Aldrich), dichloromethane (DCM, SupraSolv®, Sigma-Aldrich), 96% ethanol (EtOH, SupraSolv®, Sigma-Aldrich), and demineralized water. For the PHO batches, only the hydrophobic solvents heptane and DCM were used. The PHI batches were treated only with hydrophilic solvents: 96% EtOH and demineralized water. For each procedure 13–17 sticks were used.

To counteract losses in extraction efficiency due to the geometry of the sticks, the maximum possible number of extraction cycles was used, increasing the probability of analytes being washed out of the maze of wood cells. The extraction conditions for each solvent and batch were 9 × 5 min cycles, 90°C and 150% rinse volume, except for ethanol, for which 100°C were used. Void volume in the ASE extraction cells was filled with clean quartz sand (50–70 mesh, Sigma-Aldrich).

In order to get an estimate of the extraction efficiency of the sticks, an amount corresponding to 13–17 sticks of milled material was extracted with the same procedure. For spruce 3–3.7 g were used per sample, while 5–5.6 g were needed for larch. Void volume in the ASE extraction cells was again filled with quartz sand, with a cellulose paper separating sample and sand. The ratio between the gravimetric yields of sticks and milled material (in mg/g) was used as a measure of extraction efficiency. To test for residues in the total extract, the milled samples were additionally extracted with 95:5 acetone:water (2 cycles à 5 min, 100°C). Furthermore, in order to double check the results, 4 g of milled material from each of the trees was extracted with a control sequence adapted from Willför et al. (2003a), employing one hydrophobic and one hydrophilic solvent. Heptane was used instead of hexane for the sake of lower toxicity and the acetone step was repeated 3 times instead of 2 times.

The extracts were stored inside the pressurized extraction bottles at 4°C until further processing. The sample volume was reduced to 50 ml and 2–4 × 10 ml aliquots were used to determine the gravimetric yield. Two of the water samples were lost due to a mistake in the laboratory.

For the larch water samples, the ArGal was precipitated out of the solution in triplicates, using the procedure described in Luostarinen and Heräjärvi (2013). The precipitate of each sample was dried at 60°C over night in a ventilated oven and the weight determined.

### 2.3. Identification and Quantification of the Extracts

#### 2.3.1. Gas Chromatography

Gas Chromatography (GC) was used for the separation of individual analytes of the respective extracts. Identification was done using the response from the Mass Spectrometrometer (MS). Quantification was done based on the signal of the Flame Ionization Detector (FID) obtained from one run per solvent fraction of each clone (*n* = 2). The extracts of all milled samples were run.

Gas chromatography of the water fraction of both species did not show any peaks, likely because the majority of the material extracted by water are polysaccharides or other polymers. Thus, water-ethanol (WE) supernatant of the ArGal-free samples was pooled from 3 determinations and subsequently dried over Na_2_SO_4_ (ACS reagent, ≥ 99.0%, anhydrous, Sigma-Aldrich). After filtration and ethanol-wash of the filtrate, the now alcoholic solution was reduced to 5 ml. Only the hydrophilic extracted larch 1 WE-samples were analyzed by GC-MS, but here the extracts of the milled material and of the sticks were compared.

An aliquot of 0.5 mg/ml of each extract was mixed with 200 μl internal standard (0.2 mg/ml heneicosanoic acid—HIA and betulinol—BET, both from Sigma-Aldrich, in methyl-tertiary-butyl-ether) and derivatized before subsequent separation according to the procedures found in Nisula (2018), Willför et al. (2003b), and Zule et al. (2015). Derivatization reagents were acquired from commercial sources and used as received.

GC-MS and -FID experiments were performed on an Agilent 6890N/5973N-system (MS Consult, 2740 Skovlunde, Denmark), equipped with an S/SL inlet for sample introduction, using bleed and temperature optimized (BTO) high temperature septa and an Agilent Ultra Inert, split, low pressure drop liner with glass wool. The inlet was connected to the analytical column (HP-1, 25 m, 0.20 mm ID, 0.11 μm) by way of 5 m Agilent Ultimate Plus deactivated fused silica tubing (0.25 mm ID), used as a sacrificial pre-column. Eluents were split between the MSD for identification and the FID for quantification using an EPC pressure controlled CFT-splitter, sending 10% to the MSD and 90% to the FID. The original protocol for the GC-MS can be found in Örså and Holmbom (1994). Before and after all runs, injections of neat derivatizing reagent were used to passivate the chromatographic system. The GC-Program was as follows: Injection volume 1 μl, split 10:1. Starting temperature 120°C, heating rate 6°C /min to 325°C, hold time 4 min. Flow rate 0.9 ml He/min, solvent delay 3 min, FID data were collected at 10 Hz.

To estimate the linearity of the method over several orders of magnitude, a calibration row was run with one varying (HIA) and one constant standard (BET). The concentrations of HIA where 0, 0.005,0.025, 0.050, 0.502, 1.003, 3.010, and 5.017 mg/ml, and BET was constant at 0.55 mg/ml. The series was run once with all concentrations, and twice leaving out the 1 and 5 mg/ml samples. A linear regression based on the ratio of the two peaks was made for each of the runs (in Microsoft® Excel 2016), on a 95% confidence level. All three regressions gave an R^2^ > 99.97%.

The limits of detection (LOD) and of quantification (LOQ) were determined for each calibration row by means of the first 4 points (0–0.05 mg/g), using Equations (1) and (2), respectively.

(1) $$\begin{array}{l} LOD=3S/b \end{array}$$

(2) $$\begin{array}{l} LOQ=10S/b \end{array}$$

where *S* is the standard deviation of the y-intercepts and *b* the slope of the corresponding regression. The average of the three determinations was used as the final value. The LOD was found to be 0.004 ± 0.001 mg/ml, corresponding to 0.005–0.008 mg/g dry wood (sample size 3–5 g). The LOQ was found to be 0.013 ± 0.004 mg/ml, which corresponds to 0.015–0.025 mg/g dry wood (sample size 3–5 g).

### 2.4. Sorption Isotherm of Kurile Larch

Samples of Kurile larch were used to obtain the sorption isotherm, using two techniques: conditioning above saturated salt solutions (4 points, 64–95% RH, hygroscopic range) and the pressure plate technique (3 points, 99.64–99.99%, over-hygroscopic range). An overview of the different RH-levels and techniques used is given in Table 1.

**Table 1:** Measurement techniques and respective conditions for the determination of the sorption isotherm of Kurile larch.

**Method**	**Salt used**	**Relative humidity**	**Pressure**	**Water potential**	**Calculation of**
		**[%]**	**[bar]**	**[J kg^-1^]**	**RH % by**
Saturated salt solution	NH_4_NO_3_	64.1	42.01	-60364	Equation 30 in Mozurkewich (1993)
	NaCl	75.5	26.6	-38172	Linear interpolation of RH values at 20 and 25°C in Table 2 of Greenspan (1977)
	KCl	85.0	15.3	-21972	
	KNO_3_	94.5	5.3	-7643	
Pressure plate	n.a.	99.67	4.44*	-44.0	Rearrangement of Equation 1 in Fredriksson and Johansson (2016)
	n.a.	99.89	1.46*	-15.0	
	n.a.	99.97	0.45*	-5	

All the RH values shown for the salt solutions were calculated as detailed in the table, and the pressure as well as the water potential were determined from these. For the pressure plate method, the pressure is the pressure that was applied in the experiment (marked with *), which was then used to calculate the corresponding RH and water potential as explained in the main text.

The TOT and NAT samples of larch 1 were used to obtain the respective absorption and desorption isotherms, in both the hygroscopic and over-hygroscopic moisture ranges, covering a total of 7 points. Thus, 7 sticks with different EW-LW patterns from the NAT and TOT groups were used. However, due to limited sample availability, sorption isotherms for the PHO and PHI samples were only determined in desorption and in the in the over-hygroscopic range. Three sticks of each treatment were used. All the sticks were labeled and cut into 5–7 equally sized pieces (≈ 0.7 × 0.3 × 0.4 mm, L × W × B). Each piece of the same stick was assigned to a different RH-level (7 for NAT and Tot, 3 for PHO and PHI). Because we suspected that the sticks were not equally well-extracted in the central part as on the borders, the pieces were distributed over the levels so that one level did not for instance contain solely center pieces.

For the absorption isotherm, the samples were dried, put into individual open Eppendorf tubes in a vacuum oven at 60°C for 24 h. The oven was then allowed to cool under reduced pressure and the cooling of the samples was finalized in a vacuum desiccator. Freshly dried molecular sieves were added to each of the tubes for storage until weighing. For determination of desorption isotherms, the samples were initially water saturated. First, they were placed in round bottom flasks RH-level-wise and set under vacuum for 15 min. Degassed Milli-Q water was added to each flask using a syringe, followed by another minute of degassing with the vacuum pump. Then the samples were allowed to stand under reduced pressure for 1h, after which atmospheric pressure was re-established and they were stored in water until weighing. Due to this water saturation step, we expected that the hydrophobic extracted pieces were extracted to a certain degree—at the very least the arabinogalactan must have been affected.

Weighing before conditioning: For the water-saturated samples, the surface water of the samples was removed by rolling each piece over a wet cellulose based cloth (Wettex, Vileda, Freudenberg home & cleaning, solutions, AB, Malmö). Then, the piece was quickly placed on the balance and the mass recorded with a resolution of 0.01 mg. The dry samples were weighed inside of a tared weighing glass filled with dry molecular sieves.

For the hygroscopic range, saturated salt solutions according to Table 1 were placed in small climate boxes, equipped with RH sensors. Once the RH stabilized, the samples were introduced. The relative humidities generated by the different salt solutions were determined as detailed in Table 1.

The pressure plate technique, which gives information on the relation between the water potential and the moisture content of the material (Defo et al., 1999), was used to determine sorption isotherms in the over-hygroscopic range. In this study, a custom-built pressure plate system was used (Fredriksson and Thybring, 2019) where specimens were conditioned in the range 0.4–4.4 bar, corresponding roughly to 99–100% RH (see Table 1). The experimental procedure as described by Fredriksson and Thybring (2019) was used. All the samples were kept in the climate boxes/pressure plate cells for a period of 2 months at 20.5 ± 0.3°C.

The conversion from relative humidities to pressure (salt solutions) and vice versa (pressure plate) was done by rearrangement of Equation (1) in Fredriksson and Johansson (2016). The water potential was calculated from the relative humidities by Equation (6) in Cloutier and Fortin (1994).

Weighing after treatment: A glove box containing an analytical balance (resolution 0.01 mg) was used. For each of the humidity levels, the RH in the glove box was adjusted accordingly using a humidity generator (2500 Humidity Generator, Thunder Scientific Corporation, Albuquerque, New Mexico, USA). For the highest levels wet cloths were additionally placed in the glove box. Finally, the desorption samples were dried and weighed as described above. The final sample size after all procedures was 4–7 replicates per group.

To enable comparison between unextracted and differently extracted samples, an adjusted moisture content, *u* (g/g), was determined relative to the unextracted weight, as shown in Equation (3) below:

(3) $$u=\left(m_{\text{w}}/m_{\text{dry}}\right)*\left(1+\left[\%extractives\right]\right)$$

where m_w_ (g) is the mass of water in the specimen and m_dry_ (g) is the dry mass of the specimen, which for the extracted material was the dry mass after extraction and [% extractives] corresponds to the amount of gravimetrically determined extractives in percent; i.e., for the NAT samples, this term is zero.

### 2.5. Fungal Degradation

Cultures of European *R. Placenta*, European strain FPRL280, were cultivated on malt agar and stored at 4°C for 2 weeks. Mycelial flocks were taken from these cultures for the experiments below.

To get a view on a more advanced state of degradation and to test for the virulence of the fungus an agar-block test was made. Six sterilized NAT sticks of spruce 2 and larch 2 were horizontally placed two by two on plastic grids in agar-filled petri dishes and incubated at 23°C and 70% RH. After 8 weeks, the samples were harvested, the mycelium removed, and the wet as well as dry weight determined.

**Figure 2:**
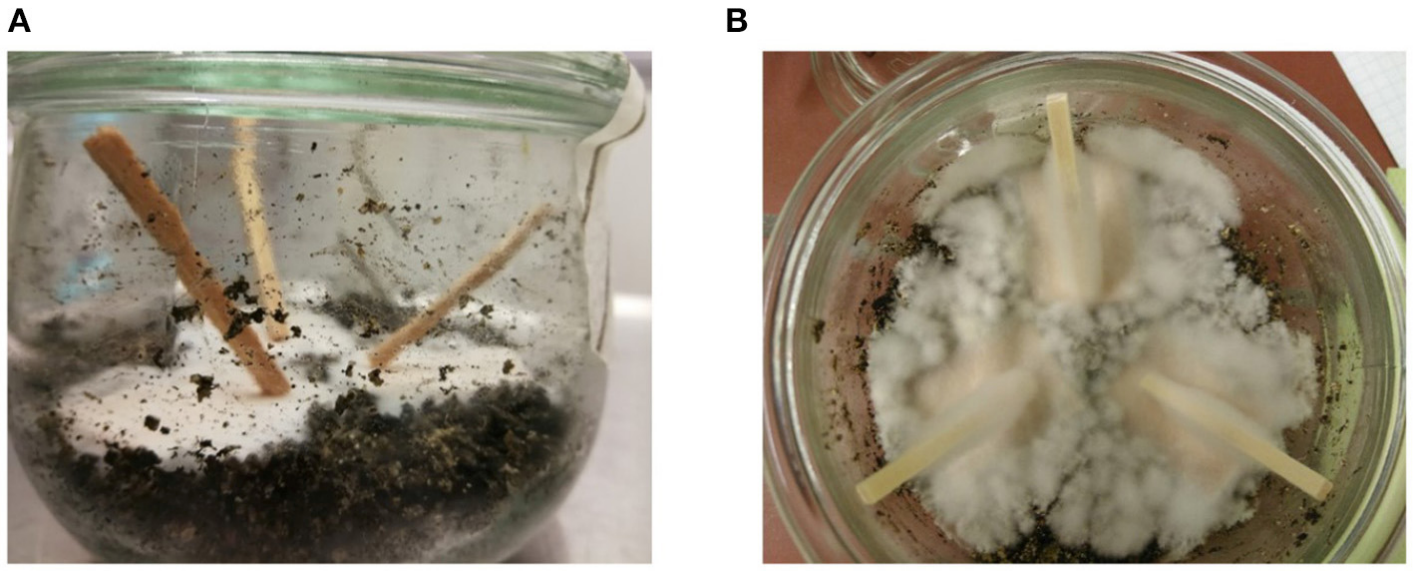
Sample setup for directional growth of *R. placenta*, inspired by Zhang et al. (2016). **(A)** Kurile larch before degradation. **(B)** Norway spruce with hyphal front after degradation.

With a very simplified version of a pole in ground-contact, we chose a modified soil-block test inspired by Zhang et al. (2016) to assess the impact of extractives on the initial phase of degradation. The fungus was allowed to grow bottom-up in the longitudinal direction of the wood for 2 weeks as shown in Figure 2. Nine sticks from each of the different extraction treatments, and native controls were marked at 2/3 of the height. The dry weight was determined, and all sticks were equilibrated at 23°C and 70% RH for 5 weeks prior to sterilization by autoclaving and inoculation. Larch 2 was additionally autoclaved before equilibration, due to suspicion of mold. The sticks were placed vertically on pre-inoculated pine feeder strips on an autoclaved soil mixture. All glasses were incubated until the hyphal front (HF) reached the marked threshold (33.3 mm). The average height of the HF in spruce was 35.5 ± 4.5 mm and was reached within 14–18 days. For larch the average HF height was 39.8 ± 5.7 mm and was reached in 15 days. Because of the geometry of the sample, there is no guarantee the hyphal front inside of the sticks reached the same height as on the outside, and thus we consider the weight loss the appropriate measure to describe the degradation. Statistical evidence for this can be found in the Supplementary Information.

After harvest, the samples were weighed immediately, stored at −25°C until all the samples had been harvested, and then dried for weight loss determination.

### 2.6. Data Analysis

#### 2.6.1. Gravimetric Yields

The gravimetric yields were analyzed in OriginPro® 2017 (OriginLab Corporation, Northamtom, MA 01060, USA, www.OriginLab.com). The standard deviations of sums and quotients of variables were calculated according to formulas taking into account statistical error propagation, i.e., formula (1) and (2) in Andraos (1996).

#### 2.6.2. Chromatograms

The mass spectral data were used to identify the peaks in the chromatograms by using a mixture of the NIST database and the database created at the Laboratory of Wood and Paper Chemistry, Åbo Akademi University. The corresponding retention times (RT) were taken from the total ion current chromatograms. For quantification of all the extracts, the peak areas of the FID-chromatogram were obtained using MSD Enhanced ChemStation^©^ (Agilent Technologies Inc., CA 95051, USA, www.agilent.com) and the amounts calculated based on the area of the internal standard peaks. Fatty acids/alcohols, resin acids, other diterpenoids, flavonoids, carbohydrates, and unknown compounds were quantified using the HIA standard, while lignans and sterols where quantified using betulinol. A correction factor of 1.2 was used for lignans, as recommended elsewhere (Willför et al., 2003a; Zule et al., 2015; Nisula, 2018; Zule et al., 2017, a.o.).

Due to the high split ratio and higher detection rate of the FID, these spectra show more resolved peaks as well as many small peaks not seen with the MS detector. The latter are quantified within the unknown (UNK) class, together with peaks that could not be identified with MS. Fatty alcohols were pooled with the fatty acids, but resin acids in different oxidation states were classified as “other diterpenoids.” Due to variations in peak intensity, some peaks below the LOD were actually detected, but disregarded from analysis. Analytes detected below the LOQ, where nevertheless included in the total sums of each chemical class.

#### 2.6.3. Estimation of Sticks Composition

Under the assumption that similar proportions of extractives were removed in sticks as in milled material, we estimated the amount of each chemical group (*i*) left in the sticks, as compared to the milled TOT samples (*f*_i, sticks_, see Equation 4). This was achieved by weighing the chromatographic yields (*y*) of each milled solvent fraction *(j*) and chemical group (*i*) with the respective extraction efficiency (*EF*). Then each chemical group was summed for each procedure and divided by the sum of yields of the milled TOT samples. For the PHO samples, the chromatographic yields obtained from the respective milled TOT ethanol fractions were added. The average of 2 trees is reported.

(4) $$\begin{array}{l} f_{i,sticks}=\overset{N}{\underset{j=1}{\sum }}y_{i,j}\times \left(1-EF_{j}\right)/\sum y_{i,j} \end{array}$$

Where *N* is the solvents relevant for the respective treatment. We have also added the remaining ArGal present in larch, which was directly calculated from the gravimetric yields, as (1-^m_sticks_^ / _m_milled__).

#### 2.6.4. Sorption Isotherm

The data were analyzed in OriginPro® 2017. Absorption and desorption data were tested individually. First a test for normality (Shapiro-Wilk) was performed for each extraction treatment and humidity level tested. For the desorption samples, all groups were normally distributed, except the groups NAT at 94% and TOT at 74% RH, hence these were excluded from follow up analysis. Among the absorption data, only the NAT at 94% group was not normally distributed, and thus excluded from further statistical analysis.

A 2-way ANOVA was performed on the rest of the desorption and absorption data, separately. The extraction treatment and the RH were used as factors, as well as the interaction terms. A power analysis was added, to test for Type II errors.

#### 2.6.5. Fungal Degradation

The statistical analysis of the fungal degradation was done in OriginPro® 2017. The variation in growth height and sampling days was tested using ANOVA as detailed in the Supplementary Information. It showed that WL was a suitable factor to describe the decay resistance.

Normality tests showed that weight loss in both species had to be log transformed for further analysis. The equality of variance was tested before the ANOVA, showing equal variance for spruce (*p* < 0.05, α = 0.05), and unequal variance for larch (*p* < 0.02, α 0.05). The normalized data were subjected to 2-way-ANOVA for each species separately, testing for tree, treatment and interaction terms. Tukey's test for comparison of means and power analysis were used *post-hoc*.

## 3. Results

### 3.1. Milled Samples: Extract Yields and Composition

**Figure 3:**
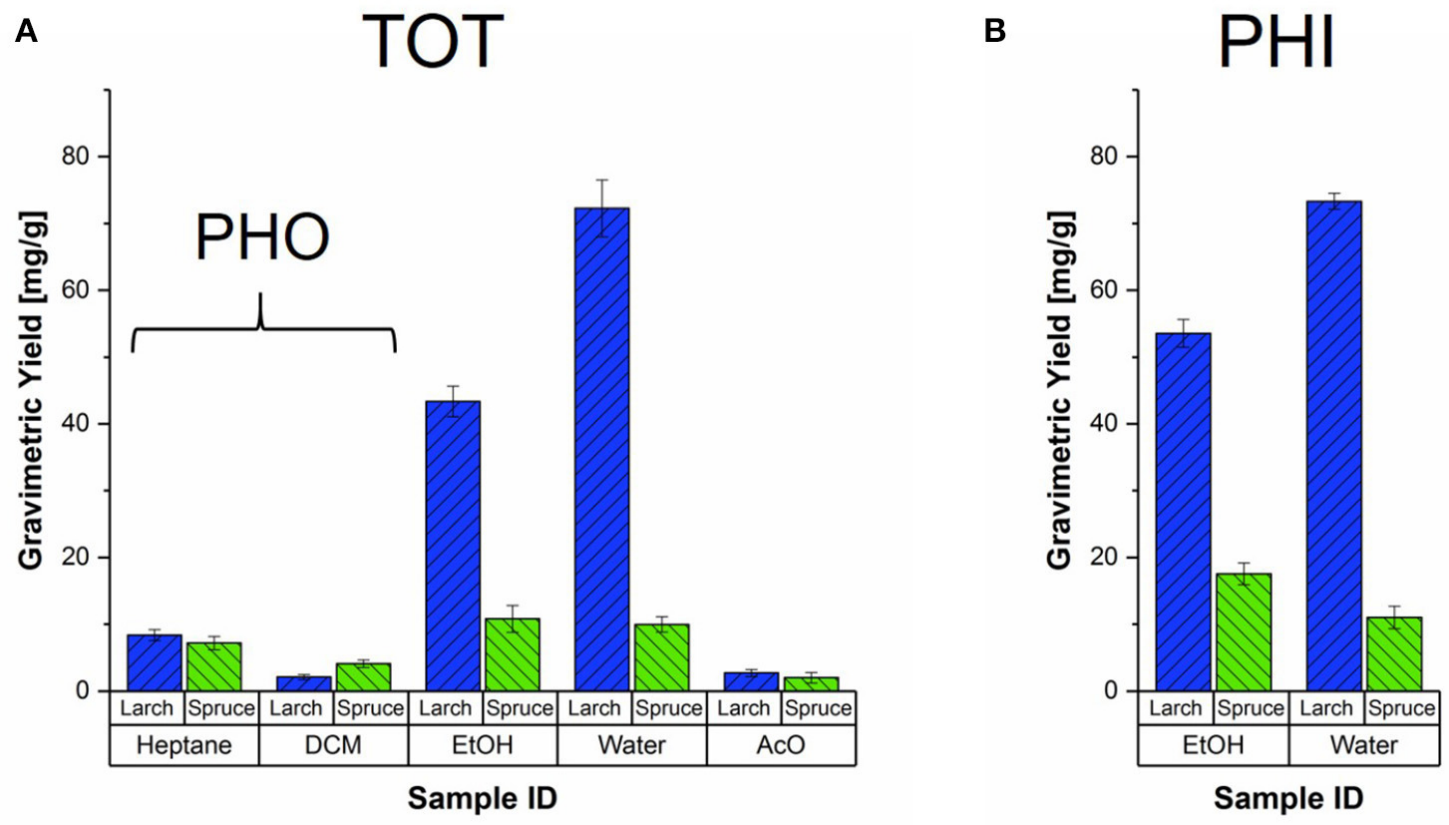
Gravimetric yields of all the solvent fractions and extraction strategies for milled Norway spruce and Kurile larch. **(A)** Average yields of the milled, total extracted samples (TOT) obtained from two clones of *Picea abies* (green) and *Larix gmelinii var. japonica* (blue), with respective standard deviations. The TOT heptane and DCM yields should be considered as reference for the hydrophobic extracted samples (PHO). **(B)** Average yields per species obtained from the extraction with hydrophilic solvents, Ethanol (EtOH) and Water.

Figure 3 shows the average gravimetric yields of the different extraction strategies of milled spruce and larch samples. The heptane and DCM fractions of the total extracted samples serve as the references for the milled PHO samples, which are therefore not shown separately. From total extraction (Figure 3A) it is evident that the amounts of hydrophobic extracts were very similar for both species, with 7–8 mg/g dry wood for heptane and 2–4 mg/g dry wood for DCM. In spruce, the yields obtained for both hydrophilic fractions were at similar levels as the heptane extracts (≈ 10 mg/g dry wood). In larch on the other hand, ethanol yielded about 5x more material on average, and the water fraction even more with about 9x higher yields, making up about 50–60% of the total sum of all solvent fractions (see also Table S1). Substantial yield differences between the two larch trees were found, reflected by the larger standard deviations (error bars in Figure 3).

When ethanol was used as the first solvent (Figure 3B), the gravimetric yields where higher than when ethanol was the third solvent. The spruce yields were slightly lower than for the sum of heptane, DCM and EtOH fractions of the TOT extraction. The average ethanol yield of the two larch trees was again much higher than for spruce, and comparable to the summed yields of the first three TOT solvents. For both species, the water fractions yielded about the same amounts as for the TOT extraction.

Upon the addition of cold ethanol to the water fractions of spruce, only very small amounts of material precipitated and could not be reliably quantified. In the case of larch, addition of cold ethanol resulted in large amounts of precipitate, known to be arabinogalactan (Côté et al., 1966; Luostarinen and Heräjärvi, 2013). Nearly all the yield of these water fractions was composed of the polysaccharide. For larch 1, the average yield of dry precipitate for the milled TOT and PHI extractions was 58.6 ± 2.7 mg/g. Reflecting the higher water extract yields, the ArGal precipitate of larch 2 amounted to 95.6 ± 3.8 mg/g on average.

To test whether the extraction was complete after the use of four different solvents, we added a fifth extraction step for the milled TOT samples. Using an acetone:water mixture (95:5), we found that in both species, 2–3 mg/g of additional material could be extracted.

In the following, the composition of the heptane and ethanol extracts of the total extraction (as assessed by GC-MS) will be described first, because the other fractions were mixtures of the latter. The composition of the additional replicates, where a control extraction procedure (heptane and acetone) was used, can be viewed in Table S2. As detailed further in the Supplementary Information, the summed yields of the individual extractive groups agreed well with the quantities obtained with the four step extraction procedure.

#### 3.1.1. Hydrophobic Extracts - Heptane

**Figure 4:**
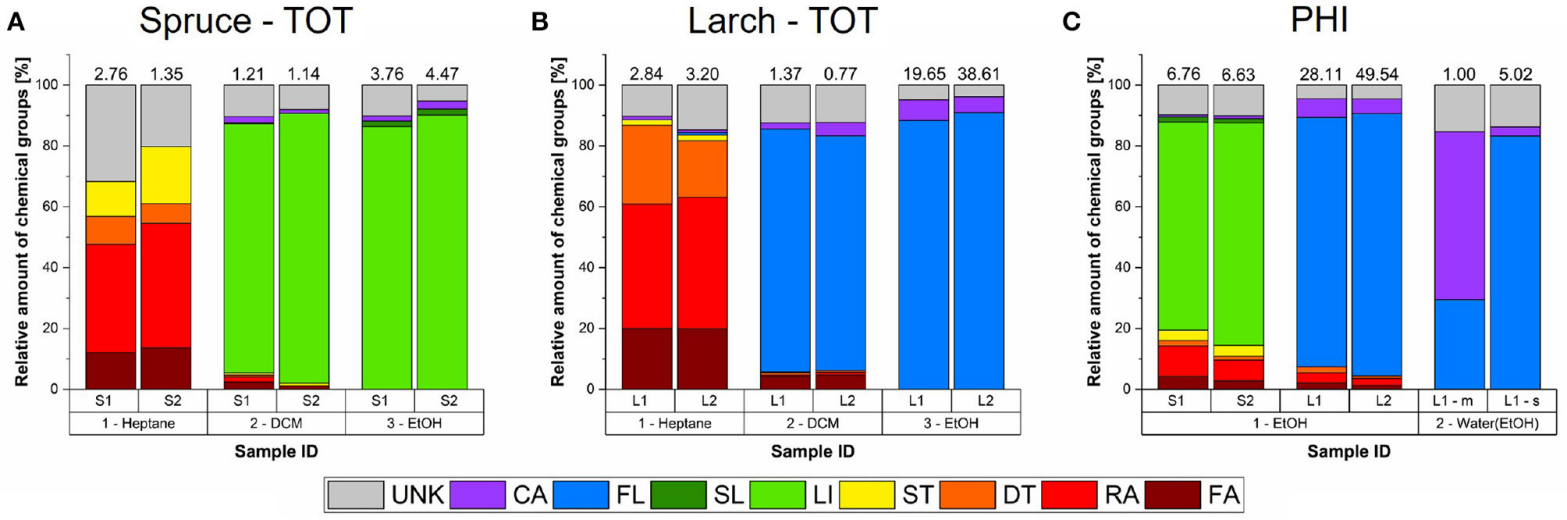
Relative chemical composition of all the detected solvent fractions and extraction strategies for milled Norway spruce and Kurile larch. For reference the absolute chromatographic yields are shown as numbers above the respective bars in mg/g dry wood (*n*=1). **(A)** Total extracted samples (TOT) of spruce (S). Each tree is shown separately, as indicated by the index. **(B)** Total extracted samples of larch (L). **(C)** Ethanol (EtOH) fractions of the hydrophilic extracted samples (PHI). The water-ethanol fractions were only run for larch 1 (sticks (s) and milled (m) extracts). Indexes before solvent designations indicate the sequence by which the solvents were used. DCM, Dichloromethane; FA, fatty acid; RA, resin acid; DT, diterpenoids; ST, sterols; FL, flavonoids; SL, sesquilignans; LI, lignans; CA, carbohydrates and other structural components; UNK, unknown.

As shown in Figures 4A,B, the hydrophobic heptane extracts of both species contained fatty acids and alcohols (FAs), resin acids (RAs), other diterpenoids (DTs), and sterols (STs), but their proportions, as well as the number of detected analytes differed. The numbers above each column in Figure 4 show the total chomatographic yield of the respective fraction in mg/g dry wood. The corresponding chromatograms can be viewed in the Figures S2, S4. The relative proportions of FAs and DTs were about 2 times higher in larch (20 and 22%, resp.) than in spruce (13 and 8%, resp.). On the other hand, spruce had 10 times higher proportions of STs (around 15%) and 3 times higher contribution from unidentified compounds (UNK, 30%). Only resin acids were found in similar proportions in both species, and where the most abundant group of hydrophobic analytes, amounting to around 40% of detected analytes. Please view Table 2 for a summary of the compounds detected using four solvents.

**Table 2:** Analytes detected in each solvent fraction of Norway spruce and Kurile larch, grouped by chemical class and the class sum (*n* = 2), as compared to the literature.

**Chemical group**	***P. abies***		***L. gmelinii var. japonica***	
	**Detected**	**Literature** **References**	**Detected**	**Literature** **References**
**Fatty acids /alcohols (FA), free**	Total: 0.20–0.36 mg/g **Palimitic acid** (16:0), margaric acid (17:0), **pinoleic acid** (5,9,12–18:3), **linoleic acid** (9,12–18:2), **oleic acid** (9–18:1), behenyl alcohol (22:0), linoceryl alcohol (24:0)	0.6–3.4 mg/g (Willför et al., 2003a) 0.25–1.4 mg/g depending on latitude, detected more types (Nisula, 2018)	Total: 0.63–0.67 mg/g palmitic acid, margaric acid, **Pinoleic acid**, **linoleic acid**, **oleic acid**, sciadonic acid (5,11,14–20:3)	1.2–3.2 mg/g, found more types (Nisula, 2018) 0.86-1.1 mg/g in *L. decidua* (Zule et al., 2015)
**Resin acids (RA)**	Total: 0.55–1.00 mg/g levopimaric acid, pimaric acid, sandarapimaric acid, isopimaric acid, **palustric acid**, **dehydroabietic acid**, abietic acid	0.48–1.5 mg/g (Nisula, 2018) 0.8–2.4 mg/g (Willför et al., 2003a)	Total: 1.17–1.38 mg/g levopimaric acid, communic acid, **isopimaric acid**, **palustric acid**, dehydroabietic acid, abietic acid, neoabietic acid	0.47–1.7 mg/g (Nisula, 2018) 4.6–7.3 mg/g (RA+DT) in *L. decidua* (Zule et al., 2015)
**Other diterpenoids (DT), free**	Total: 0.09–0.26 mg/g Thunbergol, neoabienol, only S1: palustral^*^, palustrol^*^	Present (Nisula, 2018) 0.1–1.3 mg/g (Willför et al., 2003a)^*^ RA precursors (Higuchi, 1997; Keeling and Bohlmann, 2006), were not reported in (Willför et al., 2003a; Nisula, 2018)	Total: 0.60–0.75 mg/g Thunbergol, manool (1 and 2), palustral^*^, (iso)pimarol^*^, neoabietol^*^, **cedrol**	Thunbergol - present, manool - 0.07–0.38 mg/g (Nisula, 2018) cedrol has not been reported for *Larix gmelini var. japonica* nor any other larch species^*^ RA precursors (Higuchi, 1997; Keeling and Bohlmann, 2006) (Higuchi p 258)
**Sterols (ST), free**	Total: 0.26–0.33 mg/g campesterol, **sitosterol**, sitostanol	0.24–0.36 mg/g (Nisula, 2018) 0.2–0.3 mg/g (Willför et al., 2003a)	Total: 0.05–0.06 mg/g campesterol, **sitosterol**	0.12–0.14 mg/g (Nisula, 2018) 0.14–0.18 mg/g in *L. decidua* (Zule et al., 2015) Cycloartenol was additionally found in these references (Zule et al., 2015; Nisula, 2018)
**Lignans (LI), monomeric**	Total: 4.24–5.03 mg/g Dihydroxymatairesinol, 7r-todolactol, iso-lariciresionol, todolactol A, alpha-conidendric acid, lignan a, lignan b, matairesinol, hydroxymatairesinol 1, **hydroxymatairesinol 2**, mixed lignan signal, lariciresinol, iso-hydroxymatairesinol	0.87–15 mg/g (Nisula, 2018) 0.0–2.5 mg/g (Willför et al., 2003a) secoisolariciresinol, pinoresinol and nortrachelogenin were not detected in our case (Willför et al., 2003a; Nisula, 2018)	Detected, but not confirmed: isoliovil and dihydroxymatairesinol	0.2–1 mg/g (Nisula, 2018) trace amounts (Zule et al., 2017)
**Flavonoids (FL)**	Detected, but not confirmed: naringenin, taxifolin	Trace amounts of dihydrokaempferol, taxifolin (Willför et al., 2004a)	Total: 18.45-35.73 mg/g naringenin, dihydrokaempferol, taxifolin 1, taxifolin 2	0.49-5.4 mg/g (Nisula, 2018) 15-36 mg/g in *L. decidua* (Nisula, 2018) 10-15 mg/g (FL + LI) in *L. decidua* (Zule et al., 2017)

*See Table S2 for the quantities obtained by the 2-solvent sequence. Fatty acids are given by their common names, the numbers in brackets show (position of double bond-number of carbons:number of double bonds)*.

Structures of the most abundant and/or relevant analytes are shown in Figure S1.

#### 3.1.2. Hydrophilic Extracts - Ethanol

Compared to the heptane extracts, the composition of the ethanol extracts was less complex in terms of the number of different chemical classes detected (Figures 4A,B). The chromatograms can be found in Figures S2, S4.

The spruce ethanol extracts of the TOT extraction were largely dominated by lignans (around 90%; Figure 4A), while the dominant phenolics in larch were flavonoids (also around 90%; Figure 4B). It may also be noteworthy that in both species the main phenolic compounds (bold in Table 2) were present at much greater concentrations than the other compounds, which was not the case for the hydrophobic analytes. In spruce, the difference was about 9-fold, while in larch it was much greater with over 50-fold. Note also, that the absolute amounts we found for the larch flavonoids are comparable to *L. decidua*, rather then *L. gmelinii var. japonica* as reported by Nisula (2018). Additionally, larch 2 had only one largely dominating flavonoid (90% of detected flavonoids)—taxifolin (TAX), while larch 1 had dihydrokaempferol (DHK) and taxifolin at an almost 50:50 ratio, DHK being slightly higher. The total ethanol yield was also higher for larch 2 (Figure S4). In both species, low amounts of monomeric carbohydrates and lignin components were also detected (≈ 3%), and ≈ 9% of the ethanol extracts were unknown compounds.

#### 3.1.3. Other Solvents and Solvent Sequences

In spruce, the low-yielding DMC fraction was composed of lignans to 82–88%, while 77–80% of the corresponding larch extracts consisted of flavonoids (Figures 4A,B). The rest was composed of mainly unknowns (8–12%) and residual FAs, RAs, DTs, and STs, as well as 2–4% carbohydrates and lignin monomers. The chromatograms are shown in Figures S2, S4.

Figure 4C shows the composition of the extracts obtained from the hydrophilic extraction procedure in spruce and larch was not specific for the hydrophilic compounds only. The PHI ethanol extracts of spruce (Figure 4C and Figure S3) resulted in a mixture of the analytes found in the heptane and ethanol fractions discussed above. Thus, this extract gives an overview of the proportions of GC-detectable extractives, that is 15–20% hydrophobic analytes (FAs, RAs, DTs, and STs) and around 70–75% of lignans. The rest was small amounts of carbohydrates/lignin components and unknown compounds. Similarly, the PHI ethanol extracts of milled larch were also a mixture of the heptane and ethanol fractions (Figure 4C and Figure S5). The hydrophobic analytes amounted to 4–7% of this extract, and flavonoids to 81–86% and the rest was again carbohydrates/lignin components and unknown analytes.

The water fraction of spruce did not show any peaks in the chromatograms, thus, no information on their composition is available (see the chromatogram in Figure S2). In the case of larch, removal of ArGal together with a dehydration step of the remaining supernatant, enabled the now alcoholic fraction to be successfully run on the GC. For the milled specimen of larch 1, (Figure 4C), flavonoids accounted for 55% of the yield, and carbohydrates for about 30%.

#### 3.1.4. Differences in Gravimetric and Chromatographic Yields

**Figure 5:**
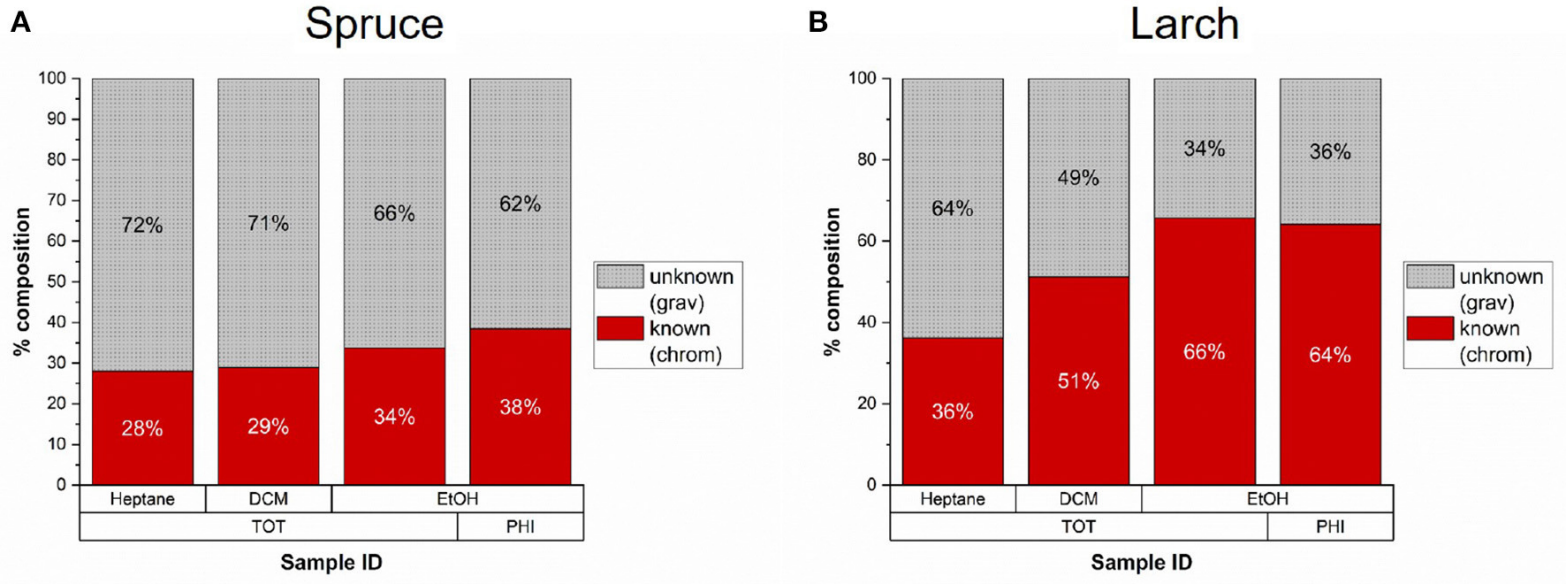
Average percentage of extractives from milled samples with known composition as determined by gas chromatography compared to the total gravimetric yield (*n* = 2). **(A)** In Norway spruce 28–38% of the gravimetric yields were detected by chromatography. **(B)** In Larch 36–66% of the extracts were detected by chromatography.

We compared the gravimetric and chromatographic yields of all milled samples in Figure 5 and found that the GC-detectable fraction varies between solvents and species. Only about 30% of the less polar spruce extracts were reliably detected with GC-FID (including unknown peaks), while up to 38% of the ethanol fractions (TOT and PHI) were detected as compared to the gravimetric yields. In case of larch, 40–50% of the heptane and DCM fractions were detected, and around 65% of the ethanol fractions (TOT and PHI).

### 3.2. Composition of the Sticks

The gravimetric yields obtained for the sticks are listed in Table S1. The resulting extraction efficiency of the sticks compared to the milled material is shown in Table 3. Note that the composition of the milled TOT heptane and DCM fractions were used for the calculation of the extraction efficiency of the PHO sticks, because we considered them representative for both groups. We found that the extraction efficiency was similar in spruce and larch. With some exceptions, the sticks yielded 50–90% for the heptane, DCM and water fractions relative to the milled material. With ethanol maximally 30–60% could be extracted from the sticks.

**Table 3:** Average extraction efficiency of the sticks that were used in the sorption isotherm and fungal degradation experiments.

**Tree ID**	**Stick/milled**	**Heptane**	**DCM**	**EtOH**	**H_2_O**
S1	TOT-s/TOT-m	0.4	0.3	0.4	n.a.
	PHO-s/TOT-m	0.5	0.5	−	−
	PHI-s/PHI-m	−	−	0.4	0.9
S2	TOT-s/TOT-m	0.8	0.5	0.6	1.1
	PHO-s/TOT-m	0.6	0.6	−	−
	PHI-s/PHI-m	−	−	0.4	1.2
L1	TOT-s/TOT-m	0.6	0.6	0.3	0.5
	PHO-s/TOT-m	0.5	1.2	−	−
	PHI-s/PHI-m	−	−	0.3	0.6
L2	TOT-s/TOT-m	0.8	1.0	0.2	n.a.
	PHO-s/TOT-m	0.6	0.5	−	−
	PHI-s/PHI-m	−	−	0.2	0.5

*The extraction efficiency was calculated as the ratio between the average gravimetric yield of sticks and associated milled sample. The yields of heptane and DCM fraction of the TOT extraction were considered representative for the respective fractions of the PHO procedure, and the calculations therefore based on the TOT yields. DCM, dichloromethane; EtOH, ethanol; S, spruce; L, larch; 1/2, tree 1 or 2; s, stick; m, milled; TOT, total extracted; PHO, hydrophobic extracted; PHI, hydrophilic extracted*.

Precipitation of ArGal from the larch water fractions of TOT and PHI sticks yielded about 40% of what the corresponding milled specimen yielded (not shown in Table 3). The chromatograms of the remaining dehydrated water extract of larch PHI sticks revealed that the composition was not the same as for the milled counterpart, as shown in Figure 4C. Flavonoids and carbohydrates were identified in both samples, but in the sticks' extract the amount of flavonoids was much higher than for milled specimen—in absolute and relative amounts—making up 80% of the yield (≈ 5 mg/g).

**Figure 6:**
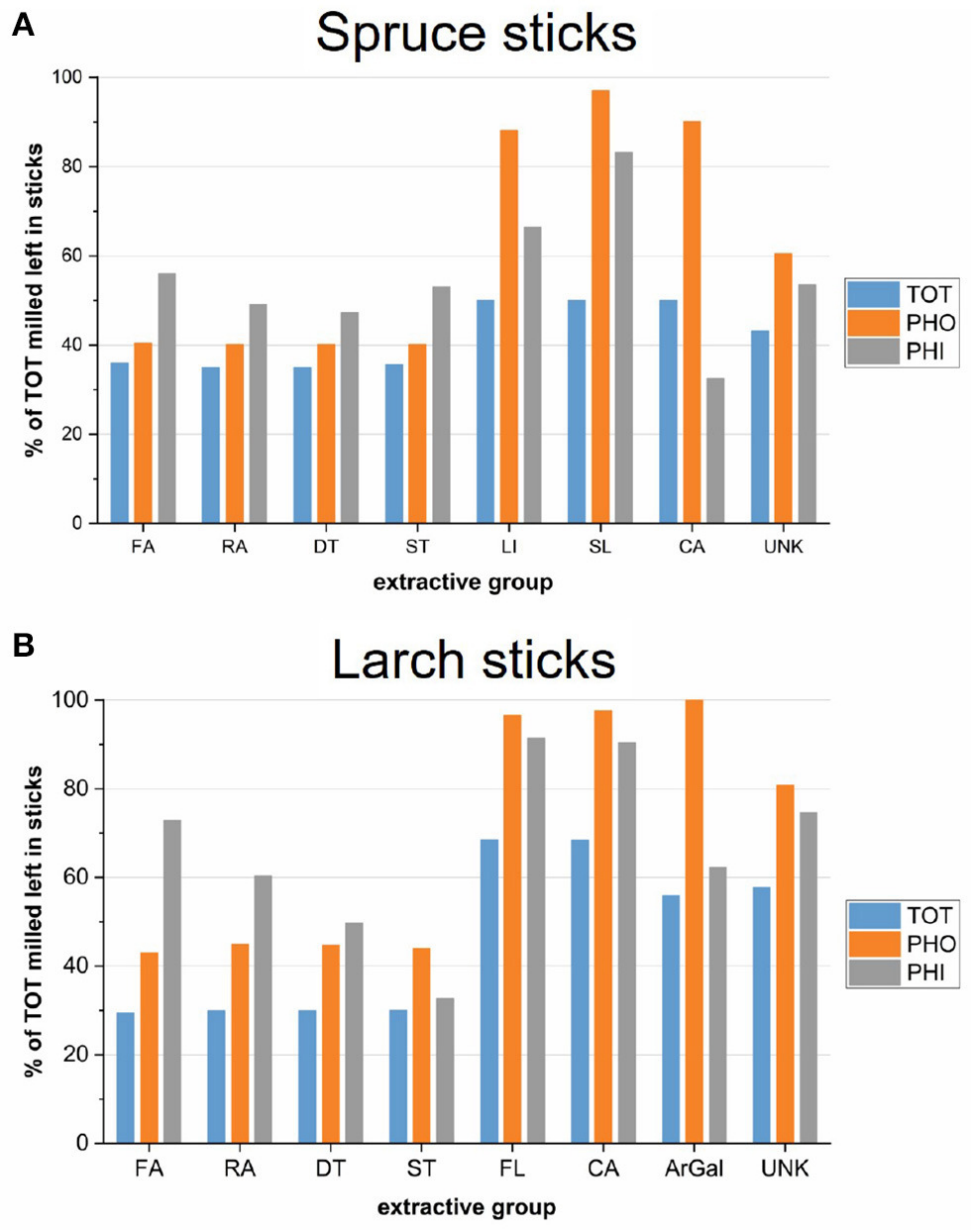
Estimates of the sticks' monomeric extractive composition after extraction: The chromatographic yields of total extracted, milled samples were weighed by corresponding extraction efficiency and extractive groups summed over the solvent fractions. The water fractions were disregarded. The average of both trees is shown, respectively. **(A)** Spruce sticks. **(B)** Larch sticks. FA, fatty acid; RA, resin acid; DT, diterpenoids; ST, sterols; FL, flavonoids; SL, sesquilignans; LI, lignans; CA, carbohydrates and other structural components; ArGal, arabinogalactan; UNK, unknown.

Despite uncertainties, we tentatively estimated the residual amounts of different extractive groups in the sticks after the different extraction procedures (Figure 6), using Equation (4). The milled TOT samples were used as a reference, because we consider it the most complete extraction. Residual ArGal was estimated based on the gravimetric yields of the precipitates.

The levels of retained hydrophobic extractives was slightly higher for the PHO extractions than for the TOT extraction in both species, even though a contribution from ethanol in the TOT procedure can be excluded, as no hydrophobic analytes were detected in this fraction. Differences may arise from experimental variations, such as the moisture content of the material upon starting the extraction. On the other hand, total extraction removed lignans (LI, SL) more efficiently in spruce (approx. 50% left), than flavonoids in larch (approx. 70% left). One reason could be that DCM extracted about 20% of the total monomeric lignans in spruce, while flavonoids in larch seem to be more polar, because DCM removed only about 1–4 % of total flavonoids.

The ethanol step of the PHI extraction removed a significant portion of the rather hydrophobic extractives (FA, RA, DT, ST), although lower amounts as compared the other extraction procedures in both species. The extraction efficiency of ethanol as the first solvent (PHI) was lower in larch than in spruce. A contributing factor here could be the presence of ArGal in larch, which is highly insoluble in alcohol, and while occupying the lumina of many tracheid cells, may reduce the flux of solvent during extraction. More than half of the polysaccharide remained in the TOT and the PHI groups (Figure 6B), although it is likely that the water extraction step relocated parts of it. As water was not used in the PHO group, we consider the relocation issue to be smaller for these samples. Nevertheless, solvent flux during extraction might be different in spruce than in larch.

The metabolic profiles described above can be summarized as follows: While the TOT samples contain the lowest amount of extractives in all categories, the PHO samples contain the highest amounts of hydrophilic extractives, including most unidentified compounds and large amounts of ArGal in larch. The PHI extraction resulted in samples having a higher proportion of hydrophobic material compared to the TOT group, but also higher amounts of hydrophilic compounds. It should thus be considered the least efficient extraction, especially in larch. A likely consequence of the incomplete extraction of the sticks is a relocation of extractives to areas where they might not be present in the native wood, which might influence their mode of action and/or efficacy.

### 3.3. Sorption Isotherm of *Larix gmelinii var. japonica*

**Figure 7:**
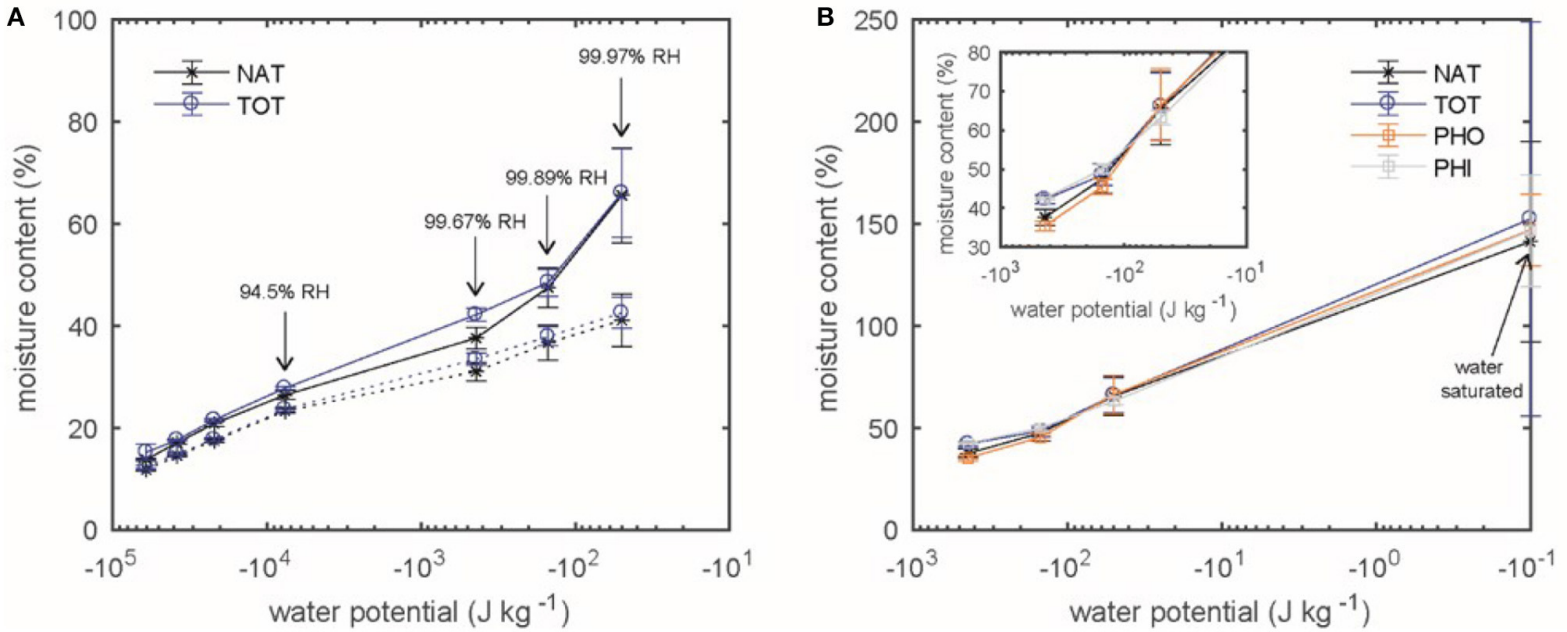
The adjusted MC (according to Equation 3) is plotted as function of water potential for Kurile Larch. **(A)** The absorption (dotted line) and desorption (solid line) isotherms for native (NAT) and total extracted (TOT) samples is shown. **(B)** Desorption isotherms in the over-hygroscopic range are shown for native larch (NAT) and after extraction with 2 hydrophobic (PHO), 2 hydrophilic (PHI) or a total of 4 solvents (TOT).

The sorption isotherm for Kurile larch is shown in Figure 7A for native and total extracted wood in the range of 64–100% RH. The MC of the TOT samples was generally slightly higher than for NAT in both absorption and desorption. The adjusted MC of the TOT samples deviated more from the NAT samples in the range of 94.5–99.89% RH in desorption mode and up to 99.97% in absorption mode. Above and below that, the curves coincide (Figure 7A). Nevertheless, 2-way ANOVA showed that this difference was only significant in absorption mode (*p* = 0.028, α = 0.05, power = 0.59) in the RH range of 64–99.97%. Also, as expected, the differences in MC at different RH-levels was significant (*p* = 0, α = 0.05).

Figure 7B shows the desorption isotherm in the over-hygroscopic range for native, totally extracted, hydrophobic and hydrophilic extracted larch. Although NAT had the lowest MC compared to the extracted samples, none of these differences were significant (*p* > 0.05, α = 0.05). Again, the difference in MC was different between the RH-levels as expected (*p* = 0, α = 0.05).

### 3.4. Fungal Degradation

The 8 week degradation test of native Norway spruce 2 and native Kurile larch 2 with *R. placenta* resulted in an average weight loss of 30.0 ± 12% for spruce (*n* = 5), while it was about half for larch, with 14.8 ± 6 % (*n* = 6). This shows that, as expected, Kurile larch was more resistant to degradation by this brown-rot fungus.

A similar pattern was found for the 2 weeks degradation test, after which a small weight loss could already be observed. For every treatment group, spruce lost close to twice the amount of mass compared to larch, except for the hydrophilic extraction, where the weight loss was almost identical for both species. The average weight loss percentage of each species and treatment is shown in Table 4.

**Table 4:** Average weight loss percentage and associated standard deviations of sticks of two clones of Norway spruce and Kurile Larch after different extraction treatments.

**Treatment**	**Spruce 1^A^**	**Spruce 2^A^**	**Average spruce**	**Larch 1^D^**	**Larch 2^E^**	**Average larch**
Native	3.7 ± 1.2^a, b^	3.3 ± 0.8^a^	3.5 ± 1.0^a^	2.5 ± 1.5^d^	1.2 ± 0.3^d^	1.9 ± 0.9^d^
Total	5.3 ± 1.5^a, c^	5.1 ± 1.3^b^	5.2 ± 1.4^b^	3.2 ± 1.0^d, f^	2.8 ± 0.8^e^	3.0 ± 0.9^e^
Hydrophobic	6.0 ± 1.9^c^	4.2 ± 0.5^a, b^	5.1 ± 1.2^b, c^	3.8 ± 1.0^e, f^	1.8 ± 0.4^d, e^	2.8 ± 0.7^e^
Hydrophilic	3.6 ± 1.1^a, b^	4.6 ± 1.7^a,b^	4.1 ± 1.4^a, c^	4.3 ± 1.2^e, f^	2.6 ± 0.4^e^	3.4 ± 0.8^e^

*For each treatment n = 18, except native of trees with index 1, where n=17. Statistically significant differences on a 95% confidence level are denoted with different letters pairs, all at p ≪ 0.001. Uppercase letters are used for the average trees (column-wise), lowercase for the treatments (row-wise); Letters “a-c” were used for spruce, “d-f” for larch*.

The average weight loss of spruce was between 3.5 and 5% over all groups. The 2-way ANOVA performed on the spruce data showed that the trees were not significantly different from each other (*p* > 0.05, α = 0.05, power = 0.15), but that the treatments were different (*p* ≪ 0.001, α = 0.05, power = 0.99), but also that the trees were differently affected by the treatments (interaction terms, *p* = 0.046, α = 0.05, power = 0.94). Tukey's test showed that the TOT and the PHO groups were more affected than the NAT group, and that the TOT group was also different from the PHI group. For the interaction terms, the same was found for spruce 1, but in the case of spruce 2 only the TOT group was different from the NAT group.

For Kurile larch, the range of weight loss was between 1.8 and 3% over all groups. The ANOVA showed that the trees were significantly different from each other (*p* ≪ 0.001, α = 0.05, power = 0.999), which can be seen from the individual weight loss of the trees, were larch 2 lost about half the weight compared to larch 1 in all treatment groups (Table 4). In fact, the weight loss recorded for larch 1 was much more comparable to the weight loss of the spruce specimen, despite having much higher phenolics content. The treatments affected the weight loss of larch differently (*p* ≪ 0.001, α = 0.05, power = 0.999) and also the trees were affected differently by different treatments (interaction terms, *p* = 0.03, α = 0.05, power = 0.96). Tukey's test revealed that the WL after all extraction procedures were different from the native group, but not from each other. For the interactions, we found that in both trees the WL of the PHI group was different from the NAT group. However, in larch 1, only the weight loss of the PHO group was higher than the NAT group, while in larch 2 the weight loss of the PHI group was higher than for NAT.

We also looked at the moisture content, calculated relative to non-degraded mass of the samples. After 8 weeks of degradation the reduced moisture content was the same for native spruce (47 ± 13%) and native larch (43 ± 9%). A similar relation is found for samples after 2 weeks of exposure to *R. placenta*, although the MC was lower. The reduced MC of 35% was again the same for all four trees, irrespective of the extraction procedure or weight loss.

## 4. Discussion

### 4.1. Extraction and Analysis

Before any further conclusions are drawn from the collected data, a few methodological issues should be acknowledged.

The performance of a living tree is yet different than what we can observe here, as we removed the majority of volatile monoterpenes during drying, which are known to play an important role in defense (Schaller, 2008, p. 164). Furthermore, despite having increased the number of extraction cycles to a maximum, the yields of the sticks were much lower than for the milled counterparts. It is questionable whether an increase in cycle duration would have significantly increased the yields further. We also found differences in the composition of the water extracts of larch 1 sticks and milled specimen. Similarly, Ekeberg et al. (2006) compared the yields and content of acetone-extracted powders and blocks of *Pinus sylvestris L*. and found that i.e., fatty acids were extracted more efficiently from blocks than resin acids or stilbenes. As we did not run the sticks' extracts on the GC, we can base our discussion only on the estimated composition of the sticks (Figure 6).

#### 4.1.1. Gas Chromatography and Polymers

We obtained a detailed view on the monomeric composition of our extracts, but noticed that, depending on the solvent, up to 70% of the gravimetrically determined mass, was not detected with the GC-MS/FID method used in this work. There can be various (cumulative) reasons for this, which will be shortly outlined below:

Low-molecular-weight compounds, especially many of the acidic extractives, are known to form esters with different alcohols, such as glycerol, sterols and other diterpenyl and aliphatic alcohols, as well as with various carbohydrates (Fengel and Wegener, 1989; Rowe, 1989; Otto and Wilde, 2001; Willför et al., 2003a). As a consequence, some compounds do not have sufficient volatility to reach the detector with the column used in this work (Zule et al., 2015).

The most significant contribution is certainly the presence of polymers, the molecular weight of which is too high for regular gas chromatography. Polymerization reactions may also occur during extraction and work-up (Holmbom, 1999, i.e., p.128), and thus introduce artifacts. Our extracts were analyzed fresh, but we saw some precipitates in the acetone extracts of both species as the samples aged. Detailed information, as well as respective countermeasures can be found in the literature (i.e., Willför et al., 2006b).

Nevertheless, most of the mass not detected with the GC-MS method used int his work is expected to be from natural polymers. The rather hydrophilic lignans and flavonoids (Willför et al., 2004b, 2006b; Fedorova et al., 2015), and more recently, also the rather hydrophobic resin acids and fatty acids (Smeds et al., 2016, 2018) have been shown to be present as oligomers and polymers. In *Picea obovata Ledeb*. from Siberia, oligomeric lignans were found to constitute up to 40% of ethyl acetate extracts (Fedorova et al., 2015). Several studies on spruce knotwood found that up to 70% of the ethanolic extracts were polymeric lignans (Willför et al., 2003a, 2004b), but also polymerized fatty acids, resin acids and other diterpenoids (Smeds et al., 2016). This agrees surprisingly well with our findings, since only 30% of the spruce extracts could be quantified by GC-FID. Therefore we suggest, that there may be similar proportions of polymeric material in the stemwood as in the knotwood of spruce, although with much higher abundance in knots (Willför et al., 2003a). Similarly in larch, Ostroukhova et al. (2012) mention a publication where the resin of Siberian larch should contain 30% of a polymer, consisting of taxifolin subunits. This again corresponds rather well with the amount of substance we were able to quantify from the ethanol/acetone extracts using gas chromatography.

Investigations of pine knotwood determined large amounts (> 50%) of polymerized fatty acids, resin acids and other diterpenoids (Smeds et al., 2018). Although, to our knowledge, such studies have not been conducted for larch, and only in knotwood of pine (Smeds et al., 2018) and spruce (Smeds et al., 2016), it may very well be that it is a property of resin in general to form polymers of its constituents. Thus, it could explain the difference in gravimetric and chromatographic yields of the heptane fractions of spruce, as well as larch. There are many techniques that allow identification and quantification of the polymeric fractions (Holmbom, 1999; Willför et al., 2003a, 2006b; Fang et al., 2013; Smeds et al., 2016; Zule et al., 2017). However, none of these options were used in the present study.

#### 4.1.2. Extractives in Spruce and Larch

Our Norway spruce specimens showed fewer analytes in some of the extractive groups than reported in literature, and slight differences between the two clones were seen. Our data from the 2-solvent sequence confirm the quantities (Table S2), and these also corresponded well with literature references (Table 2). We found relatively low amounts of fatty acids and fewer types than reported in the references (Nisula, 2018); also levopimaric acid (a RA) was not found. This might be due to natural variation with regard to genetics or the environment, or because these analytes were below the LOD of our setup, or even due to artifacts from extraction and/or work-up. Alternatively, some infection might have happened already in the live tree, which has been shown to decrease the amount of free FAs, but also deplete the amount of levopimaric acid (Ekman, 1980). The fatty alcohols lignoceryl alcohol and behenyl alcohol were also detected and are known to be the most common alcohols found in *P. abies* (Fengel and Wegener, 1989, p. 194). Due to the detection method used, we have no information about the amounts and types of esterified FAs, but they are known to be less abundant in spruce heartwood than in sapwood (Willför et al., 2003a). Among the diterpenoids, neoabienol had a higher concentration in spruce 1, explaining the different proportions of DTs among the two trees (Figure 4). The relative amounts of sterols in our spruce specimen were also different, but the absolute amounts were the same, the difference likely arising from the difference in unknown compounds. Also the lignans detected in our specimen were fewer than described in the literature (Willför et al., 2003a; Nisula, 2018), but again, some of these might have been below our LOD. The precipitate obtained from the water fraction of spruce suggests the presence of hemicelluloses, possibly of arabinogalactan type (Willför and Holmbom, 2004).

Similarly, in Kurile larch a few analytes were not detected—or detected and not found in the literature. Apart from this, the extractive profiles corresponded well with previous literature for the same (Nisula, 2018) and other species of larch (Zule et al., 2015, 2017; Nisula, 2018). As opposed to spruce, the heptane fraction of Kurile larch did not contain fatty alcohols, but triglycerides are also expected to be present (Zule et al., 2015). We found relatively large amounts of cedrol in our specimens, but could not find evidence for this in the literature for any species of larch. We do not know if this is an artifact or not. As mentioned, there was a rather important difference in the flavonoid profiles of the two larch clones. Firstly, larch 2 contained almost twice the amount of flavonoids of larch 1. Secondly, and as reported by several sources, Taxifolin 1 (TAX) was the dominating flavonoid of larch 2 (Venäläinen et al., 2006; Zule et al., 2017; Nisula, 2018), whereas in larch 1, DHK and TAX were present at a 50:50 ratio. According to literature, DHK is one of the direct precursor molecules of TAX (Winkel-Shirley, 2001), indicating that larch 1 had a problem with the conversion to TAX. The large difference in yields of the water fractions resulted in a 30 mg/g difference of precipitated arabinogalactan, being higher in larch 2. The amounts detected correspond to 6–10% w/w. These quantities are in agreement with findings from other authors and species of larch, where the amounts range from 5 to 20% (Côté et al., 1966; Luostarinen and Heräjärvi, 2013). The ArGal yields for the sticks were approximately 40–50% of the milled counterpart. After removal of the hemicellulose, the water fractions were successfully run on the GC and revealed that the water fraction was composed of carbohydrates and flavonoids, but the relative composition between sticks and milled specimens was not the same. Not surprisingly, a larger contribution of carbohydrates was found for the milled samples, but in absolute numbers this difference was not so drastic. The relatively higher release of flavonoids in the sticks was probably due to the limited extraction efficiency as compared to the milled material. Because the extraction efficiency of the ethanol fraction in spruce was higher on average (50%) than for larch (30%), we also suggest that the presence of ArGal in the lumen of tracheids might hinder the flow of solvent and extractives during extraction with solvents other than water.

Summarizing, we should keep in mind that (1) we do not know the composition of the fraction not detected by GC, (2) that we cannot say for sure whether the extracts of the sticks contain the same proportions of molecules as do their milled counterparts and (3) that extraction was incomplete for the sticks, possibly causing migration of analytes within the wooden tissue.

### 4.2. Extractives in Relation to Moisture and Fungal Degradation

#### 4.2.1. Moisture Content at Isothermal Equilibrium and After Fungal Degradation

Investigating the moisture content of the Kurile larch samples, we found that the TOT samples had a higher MC than the NAT samples in absorption, especially in the range between 94.5 and 99.89% RH. This difference, however, was not statistically significant in desorption, although we observed that at saturation (100% RH), the NAT samples had the lowest MC (about 140%) and the TOT samples the highest with 150% MC.

Previous studies, performed in the hygroscopic range, indicate that there is a difference in both absorption and desorption modes and that it is dependent on the amount of extractives (Wangaard and Granados, 1967; Choong and Achmadi, 1991; Nzokou and Kamdem, 2004; Vahtikari et al., 2017). In the hygroscopic moisture range, this observation can be attributed to a bulking effect of (polymeric) extractives, occupying space in cell walls. However, changes seen in the over-hygroscopic range where water is present also outside cell walls should rather be related to presence of extractives in pits and macro voids.

It seems that also the proportions of hydrophilic and hydrophobic extractives influence the MC in the hygroscopic range, which can be seen from the data of Nzokou and Kamdem (2004) where the resin-rich pine had a lower equilibrium MC as compared to cherry and oak at comparable extractives content. No such trend was seen from our desorption data in the over-hygroscopic range, obtained for all types of extractions of larch. Another factor may be the radial position the sample was taken from, as the MC of earlywood and latewood may differ from year to year (Hill et al., 2015). The differences in MC reported in the above mentioned references are all in the same range with the standard deviations of our findings. Therefore, it might not be so straightforward to interpret the cause of these observations and studies should be conducted to assess whether these effects can actually be distinguished from each other.

Different fungi have different moisture requirements for successful infestation of wood (Meyer and Brischke, 2015; Brischke et al., 2017). Yet, our data suggest that the extractives do not play a key role in the moisture regulation of our larch specimen, as the MC was not different enough between the extracted and native samples to explain the differences we found in degradation. This is supported by the fact that we found the same moisture content for all samples after degradation by the brown-rot, no matter what kind of extractives had been removed, and also independent of the species. It would be necessary to repeat this experiment with a non-degraded control sample, incubated under the same conditions, to see how much of that water comes from the humidity conditions inside the petri-dish and the soil-block jar. This would allow the estimation of how much water comes from fungal respiration and/or active water transport by the fungus (Thybring, 2017).

Similar to our study, it was also concluded for Scots pine (Jebrane et al., 2014) and several species of larch (Venäläinen et al., 2006; Jebrane et al., 2014) that the natural durability of wood is not necessarily determined only by its moisture content, but also by chemical properties of extractives, as will be further discussed below.

#### 4.2.2. Defense Strategy of Spruce

The weight loss of the Norway spruce samples was in the same range with Finnish spruce (Metsä-Kortelainen and Viitanen, 2009), but higher than found for Austrian spruce, which was more in range with larch specimen of the present study (Fackler et al., 2010). Despite its overall low durability, we found that spruce hydrophobic extractives play a relevant role in defense of the cell wall material against fungal attack, supported by the action of lignans. Very indicative was that the average weight loss we found for the PHO group of spruce was statistically the same as the TOT group, which both had a higher weight loss than the native samples. This suggests that the removal of the hydrophobic extractives alone had a more significant impact on the brown-rot attack, than did the additional removal of hydrophilic lignans, which are removed and/or relocated to higher extends by the PHI and TOT extractions.

We found that the major contributors to the heptane extracts were fatty acids, resin acids, some diterpenoids and sterols, out of which resin acids were dominant. These compound classes are all part of the typical oleoresin found in rays and resin channel of conifers (Higuchi, 1997), which is also used for the defense in active tissue (i.e., sapwood). Micales et al. (1994) tested pure and mixed RAs *in vitro* and detected fungitoxic effects at concentrations of 0.02% on pine wood. They noted that, generally, the abietan type resin acids (i.e., dehydro-/abietic acid, palustric acid, Figure S1.3,4) were more fungitoxic than the pimarane-types (i.e., iso-pimaric acid, sandaracopimaric acid, Figure S1.5). In our specimens the total resin acid concentration was higher than that (around 0.04%), and the most abundant analyte, dehydroabietic acid, was present at about 0.02%. The more hydrophilic lignans in the ethanol fraction exhibit relatively strong antioxidant behavior when used in higher concentrations (i.e., 2%) (Willför et al., 2003c). The total concentration of monomeric lignans of our specimens was only about 0.4%, with HMR as the main analyte. This molecule was found to be a good antioxidant *in vitro* (Rice-Evans et al., 1997; Saarinen et al., 2000; Willför et al., 2003c). In our study, resin acids were found at concentrations levels found to be fungitoxic, and lignans found at much lower concentrations than their proven effect levels. This could explain why the extraction of the hydrophobic analytes affected the spruce wood proportionally more than did the removal of lignans. Note also that spruce 1 was more severely affected, which might be due to the fact that it had a higher amount of hydrophobic substances, but a lower amount of hydrophilic ones than spruce 2. The fewer lignans in that tree could not back-up the loss of the RAs, while in spruce 2, which had a higher monolignan content, they could protect the cell wall material to a higher degree.

We saw that the TOT extraction affected the sticks samples significantly more than the PHI extraction (at least in spruce 1), but the slightly higher yield of the total extraction might not suffice to explain this difference alone. A possible additional effect could be the migration of hydrophobic molecules upon extraction, as mentioned in Nzokou and Kamdem (2004). Following this thought, and assuming more hydrophobic extractives are infiltrated in the cell wall, the extraction with heptane and DCM might have caused migration of hydrophobic substances to the cell wall surface, which were subsequently removed by ethanol extraction. In the case of the PHI samples, with ethanol as the first extraction step, the same migration may have happened, but the subsequent water extraction could not remove them efficiently, thus leaving a fungitoxic and hydrophobic layer on the cell wall surface. It was suggested that the absorption rate could be additionally reduced by such an effect and thus, potentially, early phase of fungal growth slowed down (Vahtikari et al., 2017; Sjökvist et al., 2018). Likely we were not able to see this effect because our samples were pre-equilibrated for several weeks prior to inocculation.

#### 4.2.3. Defense Strategy of Larch

For the 8 weeks degradation test, we found the same weight loss of Kurile larch grown in Denmark, as reported for European and Siberian larch of several provenances, degraded by *R. placenta* (Jebrane et al., 2014). The hydrophilic flavonoids, especially taxifolin, seemed to be the crucial ingredient to the defense of the wood via their antioxidative potential, but being supported by hydrophobic extractives. We saw that the two larch clones performed very differently in all tested groups, except in the TOT group, where the WL was almost the same. In this group the difference to the control was only significant for larch 2, indicating that the extractives it contained were more relevant to its durability than for larch 1. In contrast, larch 1 was more affected by the hydrophobic treatment, as compared to larch 2 and the controls, thus the hydrophobic extractives played a bigger role in this tree.

The phenolics content of larch 2 was about twice as high as for larch 1, while the hydrophobic extractives were present in similar amounts. This supports findings from previous studies that used the total phenolics or water-soluble extractives as a way to predict or explain decay resistance in larch trees (Scheffer and Cowling, 1966; Gierlinger et al., 2003; Jebrane et al., 2014; Nisula, 2018). Notably, the difference between the two trees lay in their respective taxifolin content, being much larger in larch 2. Venäläinen et al. (2006) also found a good correlation between the taxifolin content of several clones of Siberian larch to their weight loss. The major phenolic compounds in larch 1 were DHK and TAX at almost equal concentration, but this did not give nearly as good a protection as did TAX alone at a higher concentration in larch 2 (see Table 4). Interestingly, the only structural difference between DHK and TAX is an additional hydroxyl group at the 3' position of the B-ring (Figure S1.8) of TAX, as seen in Figure S1.9,10. It was shown that this greatly enhances the antioxidant potential in aqueous systems (Rice-Evans et al., 1996; Binbuga et al., 2008). One of the reasons is a better stabilization of the flavonoid radical when both of these positions are hydroxylated. The same configuration also increases their metal chelating abilities (Rice-Evans et al., 1996), which is important when considering that brown-rot fungi need iron to generate radicals (Binbuga et al., 2008; Ringman et al., 2014). The difference in durability of these two larch trees may thus be explained not only by the quantity, but also the increased antioxidative power, as well as metal chelating abilities of TAX over DHK.

The fact that larch 1 was more affected by the hydrophobic extraction points at the possibility that, similarly to spruce, the hydrophobic extracts are an important back-up to the more abundant hydrophilic compounds, especially when these are not so efficient. That is, larch 2 was less affected by the PHO extraction, because the high taxifolin content protected the wood well, which the mixture of TAX and DHK could not do for larch 1. Finally, the case of larch 1 gives a good example of why the taxifolin content may be a more reliable indicator for larch durability, rather than total phenolics or flavonoid content, because it was found to have a similar WL as our spruce specimen, even though the phenolics content was much higher.

#### 4.2.4. Degradation - Spruce vs. Larch

The total phenolics content is often used as a parameter in durability studies (Scheffer and Cowling, 1966; Gierlinger et al., 2003; Jebrane et al., 2014; Nisula, 2018). In the case of these two species the 4× higher phenolics content of larch, only lead to about half the weight loss within the first 2 and 8 weeks as compared to spruce. This suggests that lignans in spruce at higher concentrations could contribute to a similar or greater durability than larch flavonoids do, presumably through their antioxdiative potentials. The work of Willför et al. (2003c) supports this suggestion, as they found that the performances of spruce and larch extracts, as well as isolated compounds thereof, were similar, but not equally good at scavenging different types of oxygen radicals, or inhibiting lipid peroxidation. We suggest that the antioxidative function of poly(phenolics) in heartwood could be especially relevant in the early-phase of degradation, where brown-rot fungi disrupt cell wall polymers by employing small radicals. Although there is no quantitative information on how efficient the cellulolytic enzymes of brow-rots could be without prior oxidative disruption of the cell wall, we find it unlikely that the fungus invests so much energy into a non-enzymatic oxidation phase, if it was not necessary. Apart from the moisture content of the wood, the amount and efficacy of antioxidants may play a significant role in the on-setting brown-rot attack, because they could potentially prolong the early-phase, as is the case with larch, showing a lower weight loss as compared to spruce in equal time. Ideally, it would even starve the fungus, because it cannot access the cell wall with its enzymes.

We found that the weight loss after 2 weeks was nearly the same for the PHI groups of both species, where spruce was not significantly affected compared to their native control, but both larch trees were less durable compared to their controls. This could be related to the presence of oligolignans in spruce, that may be harder to extract and have been shown to be good radical scavengers (Willför et al., 2003c). On the other hand, the larch specimen is also expected to contain polymeric material with similar activity. A more likely reason is that the removal of hydrophilic components did not affect our spruce samples as much, because the quantities of lignans were low, and are generally low in spruce heartwood. Therefor, spruce may simply have to rely on the fungicidal oleoresin more heavily, than larch does. In this scenario, the composition of the oleoresin is crucial, and known to differ among more and less susceptible trees (Holmbom et al., 2008; Mason et al., 2015).

We tentatively conclude, that hydrophobic extractives play a more important role in spruce than in larch, and vice versa. Note though, that we cannot exclude the possibility that the observed effects were caused by a relocalization of certain substances by the extraction, to places where they may not be as effective as they would have been in their native surroundings.

## 5. Conclusion

In order to investigate the influence of hydrophobic and hydrophilic extractives on water sorption and brown-rot degradation, we prepared milled samples and sticks of Norway spruce and Kurile larch extracted with only hydrophobic or only hydrophilic solvents, as well as extracted with all four solvents (total extraction). The extraction efficiency was lowest for the ethanol fraction, indicating that ethanol-soluble analytes are harder to remove from the wood, which might be connected to their localization. For both species we found that most of the extracted material must be either polymeric or esterified in some way, because we only detected a fraction of the extracts with gas chromatography. This implies that the conclusions we draw could not consider much of the effect of the polymers in the extract, although it is known that they contribute to bulking of the cell wall. However, this effect was overall small as seen from the sorption isotherm we obtained for Kurile larch after different extractions, because of the incomplete extraction. We conclude that the changes observed are likely not responsible for the differences in degradation among the differently extracted samples.

The degradation test with European *R. placenta* resulted in measurable weight loss already after 2 weeks of incubation. We suggest that the more fungitoxic, hydrophobic substances found in spruce may play a bigger role in its protection, while on the other hand, larch durability is additionally boosted by its antioxidative flavonoid content, especially its taxifolin content. In this scenario, the hydrophilic lignans in spruce and hydrophobic extracts in larch can complement or back-up the function of the respective other part, especially in case of metabolic disturbances as likely seen with larch 1. Regarding non-toxic wood protection systems, this study indicates that large amounts of antioxidant material can provide some protection against the brown-rot *R. placenta*, but systems with multiple mechanisms of action are still preferrable.

## Data Availability Statement

The datasets generated for this study are available on request to the corresponding author.

## Author Contributions

The research was initiated and designed by SF and LT. TB-N and SF did the GC-MS/GC-FID instrument setup and measurements. AS contributed significantly to the analysis of the GC-MS data and the manuscript revisions. MF and SF did the design and execution of the sorption isotherm experiments, interpretation of data together with LT. AP helped design and discuss the fungal degradation experiments and with manuscript revisions. SF did the extractions, data analysis and interpretation of all experiments. SF, TB-N, MF, and LT co-wrote the paper. All authors read and approved the final manuscript.

## Conflict of Interest

The authors declare that the research was conducted in the absence of any commercial or financial relationships that could be construed as a potential conflict of interest.
