# Supplementary material for: Hydrophobic and Hydrophilic Extractives in Norway Spruce and Kurile Larch and Their Role in Brown-Rot Degradation
Source: Front Plant Sci. 2020 Jun 30;11:855. doi: 10.3389/fpls.2020.00855 (PMC7339921; doi:10.3389/fpls.2020.00855)
Supplement: Supplementary file 1 [file Data_Sheet_1.PDF]

Supplementary Material

Supplementary Material

Detailed working procedures can be obtained on request to the corresponding author.

The chemical structures relevant to the main text are shown in Figure S1.

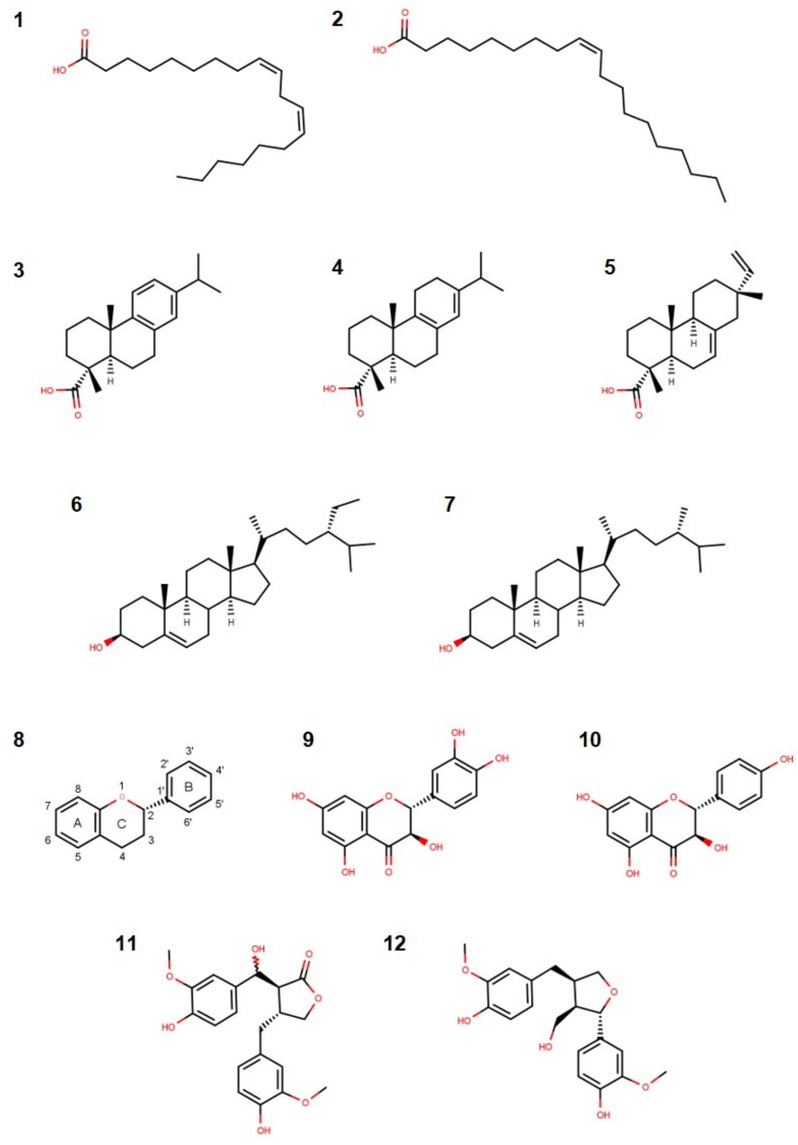

Figure S1. Chemical structures of representative analytes of each extractive family relevant in this paper. In most cases analytes relevant for the discussion were selected. Fatty acids (FAs): **1** – oleic acid ; **2** – linoleic acid; Resin acids (RAs): abietan-type: **3** – dehydroabietic acid; **4** – palustric acid; pimarane-type: **5** – isopimaric acid; Sterols (STs): **6** – sitosterol; **7** – campesterol; Flavonoids (FL): **8** – skeleton; **9** – taxifolin (TAX); **10** – dihydrokaempferol (DHK); Lignans LI: **11** – hydroxymatairesinol (HMR); **12** - lariciresinol

1 EXTRACTION

1.1 Gravimetric yields

Table S1 shows the summary of gravimetric yields obtained from each species, solvent and sequence in this procedure for the milled specimens and the sticks. The heptane and DCM fractions of the total extracted samples serve as the references for the milled PHO samples.

Table S1. The average gravimetric yields of two clones of Norway spruce and Kurile larch obtained with different extraction procedures in mg/g of dry wood (n = 4-8). Results are shown for milled (m) specimens and sticks (s). The yields of the PHO-m extraction are represented by the heptane and DCM fractions of the TOT-m group. TOT - total extracted (4-5 solvents), PHO - hydrophobic extracted (Heptane, DCM), PHI - hydrophilic extracted (Ethanol, Water). * Average yield of spruce 2 and larch 1, respectively.

Species	Procedure - Shape	Heptane	DCM	Ethanol	Water	Acetone	Sum w/o Acetone
		[mg/g dry wood]					
**Norway spruce**	TOT-m	7.2 ± 1.0	4.1 ± 0.6	10.8 ± 2.0	10.0 ± 1.1	2.0 ± 0.8	32.1 ± 2.6
	TOT-s	4.4 ± 2.0	1.8 ± 0.7	5.4 ± 2.1	9.8 ± 3.7*	-	21.2 ± 4.8
	PHO-s	4.2 ± 1.9	2.2 ± 0.5	-	-	-	6.4 ± 1.9
	PHI-m	-	-	17.6 ± 1.6	11.0 ± 1.7	-	28.6 ± 2.3
	PHI-s	-	-	6.8 ± 1.3	11.5 ± 2.3	-	18.3 ± 2.7
**Kurile larch**	TOT-m	8.4 ± 0.8	2.1 ± 0.4	43.4 ± 2.3	72.3 ± 4.2	2.7 ± 0.5	126.1 ± 4.9
	TOT-s	5.8 ± 1.1	1.6 ± 0.2	11.7 ± 1.8	30.8 ± 4.3*	-	50.0 ± 4.8
	PHO-s	4.4 ± 0.9	1.8 ± 0.1	-	-	-	6.2 ± 0.9
	PHI-m	-	-	53.6 ± 2.1	73.3 ± 1.2	-	126.9 ± 2.4
	PHI-s	-	-	12.8 ± 1.6	38.6 ± 2.5	-	51.4 ± 3.0

1.2 Chromatograms

The FID-chromatograms obtained for each solvent fraction of the TOT and PHI extractions of milled Norway spruce and Kurile larch are shown in Figures S2 - S5.

1.3 Yields obtained from alternative extraction method

Table S2 shows the chromatographic yields obtained from ASE extraction of milled material with heptane followed by acetone, adapted from [1]. This additional extraction procedure was done to compare the yields and composition to our method, as well as to other literature sources employing the same method. The same individual compounds were found as shown in Table 3 in the main text. For larch, signals of sterols were additionally seen in the FID of the acetone extracts, but these could not be confirmed, as the corresponding signals in the MS were too weak.

Table S2. Quantities of chemical groups found in milled material of clones of spruce and larch using heptane followed by acetone extraction (n=2) in mg/g of dry wood.

Quantity [mg/g]	Spruce	Larch
Fatty acids	0.20-0.21	0.48-0.60
Resin acids	0.53-0.95	1.26-1.27
Other Diterpenoids	0.10-0.23	0.47-0.81
Sterols	0.2	0.04-0.05
Lignans, monomeric	4.29-4.60	-
Flavonoids	-	25.34-34.99

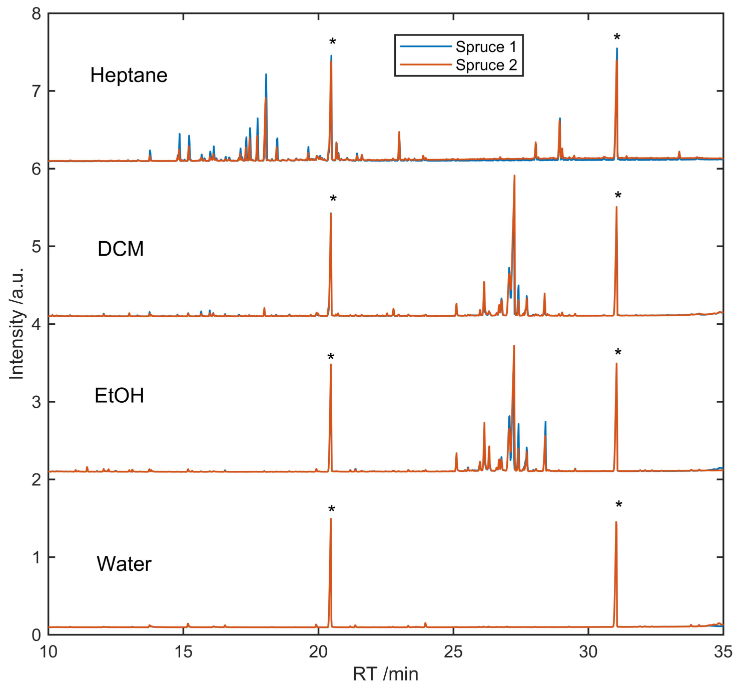

Figure S2. GC-FID chromatograms of each fraction of the total extract of Norway spruce. The chromatograms of both clones are overlaid. Peaks with asterix (*) correspond to the internal standards.

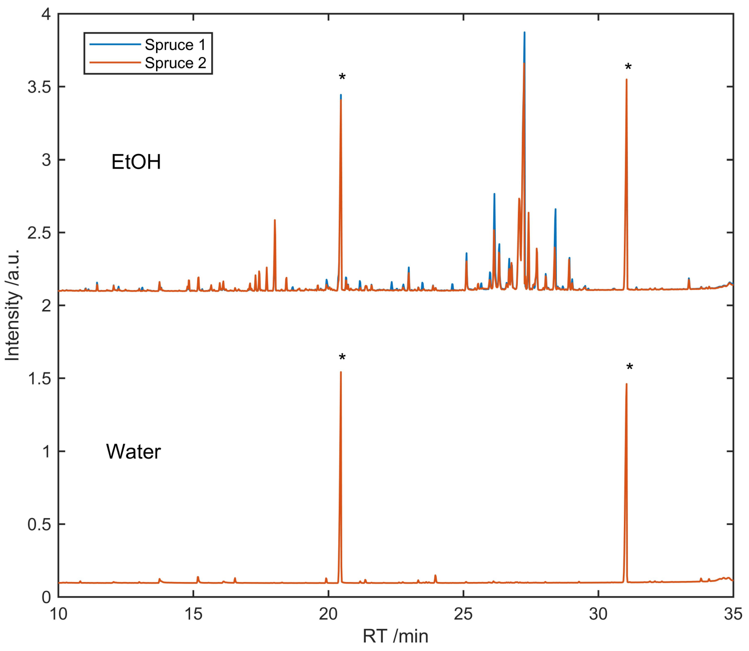

Figure S3. GC-FID chromatograms of the ethanol and water fractions obtained from the hydrophilic extraction of Norway spruce. The chromatograms of both clones are overlaid. Peaks with asterix (*) correspond to the internal standards.

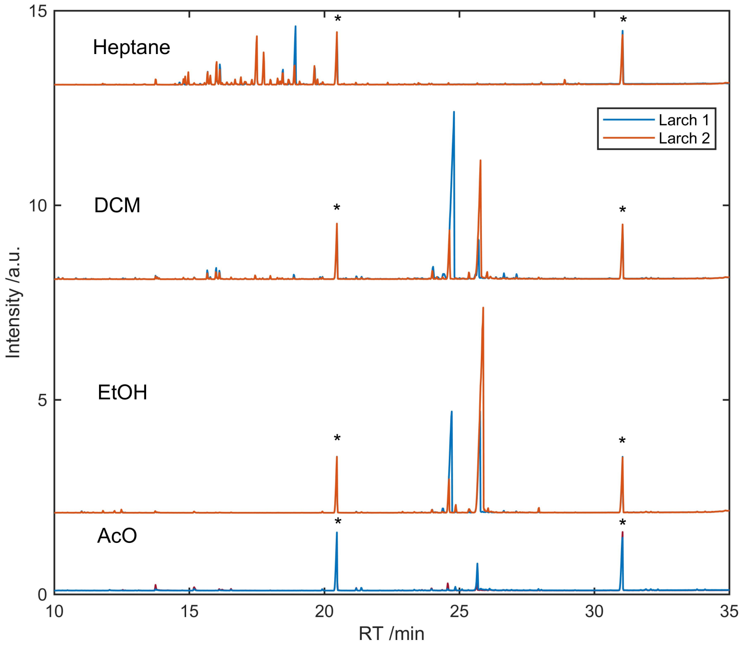

Figure S4. GC-FID chromatograms of each fraction of the total extract of Kurile Larch. The chromatograms of both clones are overlaid. Peaks with asterix (*) correspond to the internal standards.

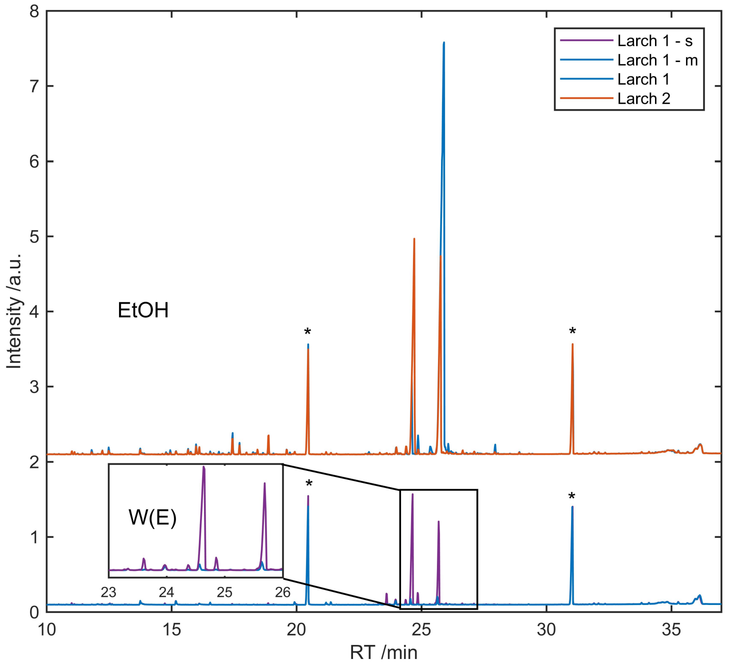

Figure S5. GC-FID chromatograms of the ethanol and water-in-ethanol(W(E)) fraction obtained from the hydrophilic extraction of Kurile Larch. For the ethanol fraction the chromatograms of both clones are overlaid. For the water fraction, the chromatograms of extracts frin sticks (s) and milled material (m) of Larch 1 were overlaid. Peaks with asterix (*) correspond to the internal standards.

2 FUNGAL DEGRADATION

2.1 No effect of harvesting time on weight loss

Due to the unpredictable growth of the fungus, apart from the treatment, two more variables potentially affecting the weight loss were considered before the analysis of the results: (1) the incubation time in days (spruce only) and (2) the height of the hyphal front (shortly called height). First, the correlation between the heights, incubation time and weight loss was asserted for each species over all treatments. Because none of the variables were normally distributed, the non-parametric Spearman correlation test was used. The Spearman correlation for spruce between WL% and incubation time was -0.22, but not significant (p>0.05, α = 0.05). Unexpectedly, the correlation between WL% and heights was even weaker with a correlation coefficient of 0.06, and not significant either (p≫0.05, α = 0.05). A possible reason for these weak and non-significant correlations is that 70 % of the spruce sticks had reached the target height and most of those that had not (85 %) were harvested on day 14. Thus, we consider the weight loss a suitable measure for assessing the effect of extraction treatment. For larch the Spearman correlation test showed a significant correlation between height and WL%, with a correlation coefficient of -0.41 and p=0.0004, α = 0.05. In this case, 60% of the sticks had overgrown the target height, and hence the height reflects weakly the weight loss. For this reason we also consider the weight loss a representative measure of the effect of treatment and will here-forth disregard the actual growth heights for the assessment of the treatment effects.

2.2 Fungal degradation by species

Figure S6 shows the average weight loss of spruce and larch after different extraction procedures.

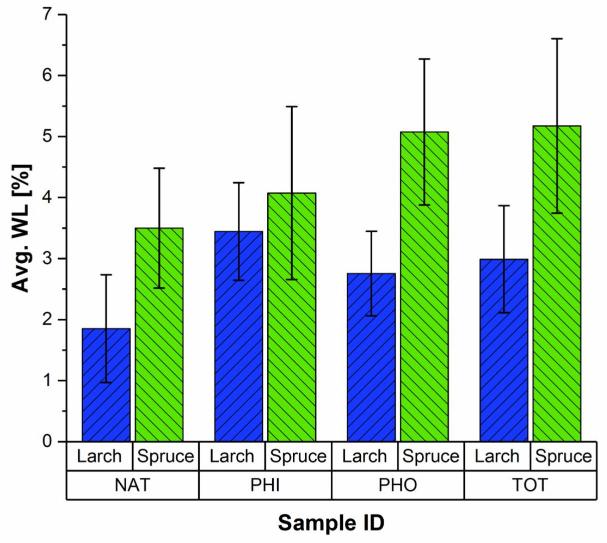

Figure S6. Average weight loss for Norway spruce (green) and Kurile larch after 2 weeks of incubation with *Rhodonia placenta*. Spruce lost twice the weight on average, for controls and every treatment group, except for the hydrophilic extraction, where larch was more strongly affected by the fungus.

REFERENCES

[1]S. Willför, J. Hemming, M. Reunanen, C. Eckerman, and B. Holmbom. Lignans and Lipophilic Extractives in Norway Spruce Knots and Stemwood. *Holzforschung*, 57(1):27–36, jan 2003.
